# Study Protocol: The Norfolk Diabetes Prevention Study [NDPS]: a 46 month multi - centre, randomised, controlled parallel group trial of a lifestyle intervention [with or without additional support from lay lifestyle mentors with Type 2 diabetes] to prevent transition to Type 2 diabetes in high risk groups with non - diabetic hyperglycaemia, or impaired fasting glucose

**DOI:** 10.1186/s12889-016-3929-5

**Published:** 2017-01-06

**Authors:** Melanie Pascale, Nikki Murray, Max Bachmann, Garry Barton, Allan Clark, Amanda Howe, Colin Greaves, Mike Sampson

**Affiliations:** 1 Norfolk Diabetes Prevention Study, Norfolk and Norwich University Hospital NHS Trust, Norwich, UK; 2 Department of Population Health and Primary Care, Norwich Medical School, University of East Anglia, Norwich, UK; 3 Health Economics Group, Norwich Medical School, University Of East Anglia, Norwich, UK; 4 Norwich Clinical Trials Unit, Faculty of Medicine and Health Sciences, University of East Anglia, Norwich, UK; 5 Norwich Medical School, University of East Anglia, Norwich, UK; 6 University of Exeter Collaboration for Academic Primary Care (APEx), University of Exeter Medical School, Exeter, UK; 7 Directorate of Diabetes and Endocrinology, Elsie Bertram Diabetes Centre, Norfolk and Norwich University Hospital NHS Trust, Norwich, NR4 7UY UK

**Keywords:** Type 2 diabetes, Diabetes prevention, Impaired fasting glucose, Non diabetic hyperglycaemia, Lay mentors, Lifestyle intervention

## Abstract

**Background:**

This 7 year NIHR programme [2011–2018] tests the primary hypothesis that the NDPS diet and physical activity intervention will reduce the risk of transition to type 2 diabetes (T2DM) in groups at high risk of Type 2 diabetes. The NDPS programme recognizes the need to reduce intervention costs through group delivery and the use of lay mentors with T2DM, the realities of normal primary care, and the complexity of the current glycaemic categorisation of T2DM risk.

**Methods:**

NDPS identifies people at highest risk of T2DM on the databases of 135 general practices in the East of England for further screening with ab fasting plasma glucose and glycosylated haemoglobin [HbA1c]. Those with an elevated fasting plasma glucose [impaired fasting glucose or IFG] with or without an elevated HbA1c [non -diabetic hyperglycaemia; NDH] are randomised into three treatment arms: a control arm receiving no trial intervention, an arm receiving an intensive bespoke group-based diet and physical activity intervention, and an arm receiving the same intervention with enhanced support from people with T2DM trained as diabetes prevention mentors [DPM]. The primary end point is cumulative transition rates to T2DM between the two intervention groups, and between each intervention group and the control group at 46 months. Participants with screen detected T2DM are randomized into an equivalent prospective controlled trial with the same intervention and control arms with glycaemic control [HbA1c] at 46 months as the primary end point. Participants with NDH and a normal fasting plasma glucose are randomised into an equivalent prospective controlled intervention trial with follow up for 40 months. The intervention comprises six education sessions for the first 12 weeks and then up to 15 maintenance sessions until intervention end, all delivered in groups, with additional support from a DPM in one treatment arm.

**Discussion:**

The NDPS programme reports in 2018 and will provide trial outcome data for a group delivered diabetes prevention intervention, supported by lay mentors with T2DM, with intervention in multiple at risk glycaemic categories, and that takes into account the realities of normal clinical practice.

**Trial registration:**

ISRCTN34805606 (Retrospectively registered 16.3.16)

**Electronic supplementary material:**

The online version of this article (doi:10.1186/s12889-016-3929-5) contains supplementary material, which is available to authorized users.

## Background and rationale

The number of people with T2DM worldwide quadrupled between 1980 and 2014, from 108 to 422 million [1] with international ambition aimed at holding the age standardised T2DM prevalence at 2010 levels [2]. Landmark clinical trials suggest that intensive lifestyle (diet and physical activity) intervention in a research setting reduces the risk of T2DM in highest risk groups [3–5]. These clinical prevention trials have largely focused on intensive lifestyle interventions in people at highest risk, usually with a plasma glucose (fasting or during an oral glucose tolerance test) or glycosylated haemoglobin (HbA1c), that is elevated, but not to any diagnostic threshold for diabetes [3–5]. In these trials the effect size is consistent [4, 5], and there may be long term beneficial legacy effects of the trial intervention, even after the intervention is withdrawn. Despite this, there remain concerns about the translatability of this trial evidence into normal clinical practice at scale, partly because of the very high costs associated with individualized intervention, and smaller effect sizes in ‘real world’ interventions [6–8]. These concerns were valid when the NDPS programme commenced in 2011 and remain valid now, despite the launch of the NHS England diabetes prevention programme [9]. The costs of diabetes prevention intervention can be reduced by group based delivery [6] and the team who delivered the landmark study in this area recognised that ‘community health workers’ could be an attractive model for delivering lifestyle intervention at reduced cost [6]. There has been growing interest in this model over the last few years for the clinical management of Type 2 diabetes, and to a lesser degree in diabetes prevention [10–12]. Recent changes in the diagnostic criteria for diabetes, with a shift from glucose based criteria to one based on HbA1c, with a diagnostic threshold of an HbA1c ≥48 mmol/mol is widely supported and adopted [13, 14]. However, this policy generates a large new population with prediabetes, usually defined as HbA1c ≥42–<48 mmol/mol. NICE PH38 policy guidance [15] is that these patients should receive intensive lifestyle advice and annual HbA1c monitoring, but there is relatively little trial evidence that this leads to a reduced T2DM incidence in this population [6–8]. There is still an active debate about the best approach to find those at highest risk of developing T2DM, and which glycaemic category should receive a clinical or research diabetes prevention intervention [6]. Glucose tolerance tests (GTT) are time consuming, unsuitable for mass population screening in ordinary primary care, and are unpopular with patients clinically and in clinical trials [16, 17]. Identifying those at highest T2DM risk for a trial intervention that translatable into normal clinical practice, must reflect the usual approach in normal clinical practice, which for some time has been based on HbA1c and fasting plasma glucose data. However, most of the large trials have examined populations defined as at risk using a GTT [6–8]. One final issue is that case finding for those at highest risk for entry to prevention trials or clinical prevention programs using widely available risk prediction algorithms may not be easily applicable in all primary care as some of the key variables in these risk prediction tools may be unavailable [18]. Mass screening in primary care for high risk individuals should reflect existing primary care models and available data, with limited additional workload.

## Need for trial

The aim of this 7 year NIHR funded programme [PGfAR RP – PG - 0109-10013] is to test the primary hypothesis that our novel diet and physical activity intervention reduces the risk of T2DM in a screened target population of UK adults with high risk of progression to type 2 diabetes (T2DM). The programme comprises a large screening and risk identification study, and a suite of randomised controlled trials designed to assess the effectiveness of a realistic diabetes prevention intervention, that is community based, with recruitment through general practice. The trials are based in the realities of current NHS care, with interventions delivered in groups in the local community, in part by lay mentors with T2DM, with minimal impact on general practice. The programme recognises the current complexity of screening and glycaemic categorisation of patients at risk of diabetes, including screen detected T2DM.

## Specific objectives and hypotheses

The NDPS programme has several elements: a screening programme [Project 1] to identify those at highest risk of T2DM on general practice databases in the East of England. The main element of the programme is then a randomised controlled prospective three - arm trial in those participants found to be at highest risk [Project 2], with one intervention arm supported by lay mentors [Diabetes Prevention Mentors; DPM] who have T2DM themselves. Project 3 is the recruitment, training and retention of these DPM. Project 4 ia a randomised controlled prospective three - arm trial of the same intervention[s] in people found to have a new diagnosis of screen detected T2DM in Project 1. Project 5 is the health economic analysis of these projects. Finally, Project 6 is a randomised controlled prospective two -arm pilot intervention in participants found in Project 1 screening to have non - diabetic hyperglycaemia (NDH) but with a normal fasting glucose in Project 1.

### Project 1 objective

To screen 10,000 participants at high risk of T2DM for non-diabetic hyperglycaemia [NDH; HbA1c ≥42 to <48 mmol/mol] or impaired fasting glucose [IFG; ≥ 5.6 to <7.0 mmol/l] to identify participants for randomisation into one of a suite of randomised clinical trials.

### Project 2 hypothesis

The cumulative incidence of T2DM will be significantly lower after 46 months in a high risk group with impaired fasting glucose [IFG; fasting plasma glucose ≥6.1 to <7.0 mmol/l] *or* with non -diabetic hyperglycaemia [NDH; HbA1c ≥42 to <48 mmol/mol] and an elevated fasting plasma glucose [≥5.6 to <6.1 mmol/l] randomised to our lifestyle intervention [standard intervention group], compared to a group randomised to this intervention enhanced by additional lay diabetes prevention mentor [DPM] input, or to a control group who do not receive any trial intervention.

### Project 3 hypothesis

We can identify, recruit, train and retain 70 volunteers with known T2DM to become diabetes prevention mentors [DPM] to support one intervention arm in each of Projects 2 and 4.

### Project 4 hypothesis

Mean HbA1c [as a measure of glycaemic control] will be significantly lower after 46 months intervention in participant groups with newly diagnosed screen detected T2DM [fasting plasma glucose >7.0 mmol/l and/or HbA1c >48 mmol/mol] randomised to the same intervention groups as in Project 2 than in an equivalent control group who do not receive this trial intervention.

### Project 5 objective

Health economic analysis of all aspects of the programme.

### Project 6 objective

To observe changes in mean HbA1c after 40 months in participants with non - diabetic hyperglycaemia [NDH; HbA1c ≥42 to <48 mmol/mol] and a normal fasting plasma glucose [<5.6 mmol/l] randomised to an observational controlled trial of our standard intervention compared to a control group not receiving this intervention.

Allocation into trial by glycaemic category is summarized in Table 1

**Table 1:** Glycaemic category [based on concordant paired baseline results] in project 1 and associated randomisation into trial

Diagnosis	Confirmed diagnosis based on concordant paired results	Randomised to Project
Normal result	Fasting plasma glucose <5.6 mmol/l *and* HbA1c <42 mmol/mol.	No Trial
Impaired fasting glucose [IFG]	Fasting plasma glucose ≥6.1–<7.0 mmol/l *and* HbA1c <48 mmol/mol	Project 2
Non diabetic hyperglycaemia [NDH]	HbA1c ≥42–<48 mmol/mol *and* fasting plasma glucose ≥5.6 to <6.1 mmol/l.	Project 2
Impaired glucose tolerance [IGT]	Fasting plasma glucose <7.0 mmol/l and a 2 h OGTT result of ≥7.8 and <11.1 mmol/l	Project 2^a^
Type 2 diabetes [T2DM]	HbA1c ≥48 mmol/mol *and/or* fasting plasma glucose ≥7.0 mmol/l	Project 4
Non diabetic hyperglycaemia [NDH]	HbA1c ≥42 to <48 mmol/mol *and* fasting plasma glucose <5.6 mmol/l .	Project 6

^a^IGT: participants with IFG (≥6.1–<7.0 mmol/l) *and* NDH (≥6.1–<7.0 mmol/l) were invited to undertake an OGTT between 2011 and 2013 for diagnostic reasons to exclude T2DM based on OGTT criteria before UK adoption of HbA1c diagnostic criteria for T2DM [15, 16], but not as a randomisation category

## Methods and design﻿: Description of intervention trials design

**Project 2** is a unblinded three - arm randomised parallel group 46 month clinical trial with randomisation of the target population at risk of T2DM (Table 1) to *either* a control group who do not receive the research intervention, *or* an intervention group who receive the 46 month diet and lifestyle intervention described below, *or* an intervention group who recieve the 46 month lifestyle intervention enhanced with additional direct contact from DPM.

**Project 3** is a recruitment project for DPMs to service one intervention arm of Project 2. The DPM aid in the delivery of the intervention, but are themselves studied with additional observational baseline and prospective clinical and biochemical assessments at prespecified time points during their involvement.

**Project 4** is a three - arm randomised parallel group 46 month prospective clinical trial with randomisation of the target population with screen detected T2DM (Table 1) to *either* a control group who do not receive the research intervention, *or* an intervention group who receive the 46 month diet and lifestyle intervention described below, *or* an intervention group who recieve the 46 month lifestyle intervention enhanced with additional direct contact from trained volunteers with T2DM [DPM].

**Project 6** is two arm randomised parallel group 40 month prospective pilot clinical trial with randomisation of the target population (Table 1) to *either* a control group who do not receive the research intervention, *or* an intervention group who receive the standard diet and lifestyle intervention described below.

## Randomisation and allocation

Project 2 and 4 are powered to detect the primary outcome at end point (see below), but Project 6 is a pilot controlled trial intended to inform the design of future intervention trials as little evidence exists currently to allow power estimates in this glycaemic category. Randomisation of participants is conducted automatically using a dedicated function in the trial data management system. The randomisation mechanism for Project 2 consists of a pre-prepared random list of codes [for the Intervention and control groups] that are stored in the trial database. To reduce the risk of predicting the next allocation while maintaining a reasonable even spread of intervention and control patients, the list is constructed of blocks of 17 codes [3 Control, 7 intervention and 7 Intervention + DPM]] to approximate the proportions of 170:390:390 respectively. The list was built prior to the start of the programme using standard Microsoft tools. Randomisation is asymmetric to deliver the sample sizes described. Project 4 and the Project 6 pilot Project are randomised using the same method in the trial data mangement system. Project 4 are randomised to a 1:1:1 ratio and the Pilot Project is randomised to a 1:1 ratio to control or standard intervention only.

## Methods: participants, interventions and outcomes

### Study setting and infrastructure

The programme is hosted by the Norfolk and Norwich University Hospital NHS Trust, Norwich UK, and NDPS programme staff work from the Norwich Clinical Research and Trials Unit [CRTU] at the University of East Anglia [UEA] Norwich. The programme is supported by the Clinical Trials Unit (CTU), Norwich. The programme works with 135 general practices in the East of England [Norfolk, Suffolk and North East Essex] to screen and recruit into Project 1, and the programme screens and delivers the interevention at 8 sites in existing NHS and University facilities, across the East of England.

### Participant eligibility criteria: primary care relationships and contact (Tables 2 and 3)

**Table 2:** Inclusion and exclusion Criteria for Project 1 screening and all intervention projects

Inclusion criteria	Exclusion criteria
Age 40 years or over and at least one of the below risk factors: Body mass index (BMI) ≥30 kg/m2 Parent, sibling or child with T2DM Personal history of coronary disease Previous history of gestational diabetes OR Known impaired fasting glucose, impaired glucose tolerance and/or HbA1c NDH range	Not able to provide GP details i.e. not registered with a GP or unwilling for their GP to be contacted Unable to give informed consent due to lack of capacity through severe mental health, learning difficulties or significant cognitive impairment Self-reported conditions which could adversely affect the trial results or patient clinical wellbeing such as: i. Terminal illness. ii. Antipsychotic medication, which may affect glucose tolerance iii. High dose oral steroids [>4 weeks or >7.5 mg] iv. Active treatment for malignancy v. Stage IV renal impairment or ongoing renal dialysis vi. Pregnant or lactating vii. Stage IV NYHA cardiac failure Taking part in any research study which involves a dietary or lifestyle change intervention [exceptions are participants in observational research studies] Participation in other research studies are assessed on an individual basis
	Inability to attend or comply with the interventions or follow-up scheduling
	Living with or related to someone in the programme team
*Diabetes Prevention Mentors inclusion criteria* *Age 18 years or over* *Diagnosed with T2DM for ≥2 years*	GP/clinician advice on health grounds that participant should not take part or be contacted Not able to provide GP details, not registered with a GP or unwilling for their GP to be contacted Unable to give informed consent due to lack of capacity through severe mental health, learning difficulties or significant cognitive impairment Self-reported conditions such as: i. Terminal illness. ii. Active treatment for malignancy iii. Stage IV renal impairment or ongoing renal dialysis iv. Pregnant or lactating v. Stage IV NYHA cardiac failure

**Table 3:** Coding criteria for General practice Electronic Health Record [EHR] database searches

*Search criteria*	*Coded criteria for EHR*
*Search 1*	Age ≥50 years and ≤80 years and a BMI ≥30 kg/m2 OR^a^ Age ≥50 years and ≤80 years and a weight [men] ≥93 kg & weight [women] ≥78 kg [if no height recorded]
*Search 2*	Age ≥50 years and ≤80 years and a BMI of ≥25 kg/m2 with at least one or more of the following: Parent, sibling or child with T2DM Personal history of coronary disease Previous history of gestational diabetes Known history of non-diabetes hyperglycaemia OR^a^ Age ≥50 years and ≤80 years and a weight [men] ≥77 kg & weight [women] ≥65 kg with least one or more of the following: Parent, sibling or child with T2DM Personal history of coronary disease Previous history of gestational diabetes Known history of non-diabetes hyperglycaemia
*Search 3*	Age ≥40 years with one of the following Known impaired fasting glucose [IFG] or impaired glucose tolerance [IGT] Fasting glucose range of ≥6.1–≤6.9 mmol/l
*Search 4*	Age ≥40 years with one of the following Fasting glucose range of ≥5.6–≤6.0 mmol/l inclusive HbA1c range of ≥42–47 mmol/mol inclusive

^a^In the case of no BMI record, these body weights (kg) would give a BMI > 25kg/m^2^ or > 30kg/m^2^ based on an assumed UK national average height (m)

The population of Norfolk in 2011 was 891,100 with a projected population of 935,800 by 2018 and 60% are more than 40 years old, with 5.7–9.4 per cent of the total from an ethnic minority group depending on the chosen definition. Current data suggest that the targeted Norfolk population is approximately 550,000 aged ≥40–≤75, registered at 137 GP practices within five Clinical Commissioning Groups [CCG’s] throughout Norfolk. The targeted central Ipswich (Suffolk) population has a registered population of 125,374 patients aged ≥40–≤75 in 32 GP practices in one CCG and the targeted North East Essex area [largely central Colchester] has an estimated population of 171,545 patients aged ≥40–≤75 in 38 GP practices in one targeted CCG. NDPS have contacted 207 GP practices within these seven CCG’s, providing the programme with a total population of 846,919 potentially eligible participants. All GP practices are invited to particpate and the GP practice’s electronic health record [EHR] software such as SystmOne and EMIS [The Phoenix Partnership TPP, TPP House Horsforth Leeds, UK and EMIS Health Rawdon House, Yeadon, Leeds, UK] is interrogated for patients eligible for Project 1. EHRs are real-time, patient-centred records that make information available instantly and securely to authorised users and make extensive use of Read codes and digital information is searchable via these codes. The GP practice is provided with the coding criteria based on the study inclusion/exclusion criteria (Tables 2 and 3). Patients are then written to directly from the practice using Docmail, which allows mass mailing by uploading and merging the generated patient list with the NDPS mail pack documents. The system allows the practice to securely upload the NDPS document template and mail to patients via a secure web portal. The use of Docmail for such purposes is permissible under guidance from both the Information Commissioner’s Office [ICO] and the Department of Health [DoH] UK, subject to the provisions of the UK Data Protection Act 1998. The NDPS mail pack consist of a patient invite letter and the study Participant Information Sheet [PIS]. Docmail processes the mailing requirements via their website software and is delivered by CFH Docmail LTD [CFH Practice Index Ltd, London, UK. http://www.docmail.co.uk. Company Reg. No. 09018867]. In accordance with ‘best practice’ security features to protect patient data and in compliance with the UK Data Protection Act 1998, no data are released to the NDPS team. Following this mail out interested patients contact the study team by phone and a programme administrator checks study eligibility with the potential participant via a detailed telephone screening interview prior to booking the screening appointment (Table 4) Recruitment through GP database interrogation accounts for 90.7% of programme recruitment. Self and direct GP referrals can be made via the NDPS website (http://www.norfolkdiabetespreventionstudy.nhs.uk) or via a central study telephone line.

**Table 4:** Registration details on initial contact prior to screening

Date of birth
Gender
Height
Weight
Calculated BMI
Known first degree relative with T2DM
Known personal history of coronary disease
Known history gestational diabetes
Known previous result of IFG or IGT, prediabetes or Non-Diabetic Hyperglycaemia
GP surgery address
Participant’s address
Recruitment route
Screening and intervention preferred site
Medication
Medical history

## Recruitment of Diabetes Prevention Mentors [DPM]

A central and novel part of this programme is the recruitment of people with established T2DM on GP registers who provide peer support by acting as lifestyle mentors to the participants in the intervention plus DPM arm of the programme. The DPM work with Diabetes Prevention Facilitators [DPF] to deliver the enhanced intervention. Each participant receives a 15 min semi-structured telephone call from the DPM every 4 weeks during the education session phase [first 12 weeks of programme] and then every 8 weeks between maintenance sessions. Before the newly recruited DPM’s are offered their role they are formally interviewed by the Senior Intervention Research Associate and the Programme Manager/Principal Investigator, with possible assistance from a Diabetes Patient Champion [a lay person with diabetes who represents the diabetes community]. DPM’s are recruited following a successful Disclosure & Barring Service [DBS] check and two satisfactory references. If successful the DPM is given an NHS honorary volunteer contract and receives specific training for their role. DPM are recruited via GP database recruitment searches using coding criteria (Table 3). Once a participant is randomized into a DPM intervention group, their availability for receiving calls is reviewed (NM) and assigned a DPM based on the DPM availability to make calls. This allows for the greatest possible call connnection rate.

## Training of Diabetes Prevention Mentors (DPM)

The NDPS specific standardised training programme for DPM (Table 5) is delivered in seven 120–150 min sessions over a minimum of 4 weeks to allow time for self-reflection and reading between each session. The training has two clear aims 1] to provide up to date information on physical activity, diet, pre diabetes.and the impact of lifestyle on the the progression to T2DM and 2] to practice (using role play) the key skills required. To successfully complete the group training seminars and be cleared to work with study participants, DPMs are required to attend at least six of the seven sessions (with sessions 3, 4, 5, 6, and 7 requiring compulsory attendance). Furthermore, DPMs needed to demonstrate an understanding of the intervention theory and to demonstrate successfully the active listening skills required. During the seminars, the senior research associate (NM) assesses the strengths and learning needs of each trainee and structures their following one to one practice training accordingly. The one to one practice work consisted of telephone call/s where DPMs adopt the role of the Mentor and the Senior Research Associate adopts the role of a trial participant. These calls model particular situations where it is felt the DPM needs to be ‘tested’.

**Table 5:** Summary of diabetes prevention mentor (DPM) training seminars content and aims

*Training setting*	*Seminar number*	*Seminar title*	*Session length*	*PowerPoint presentation*	*Practice work*	*Practice work aims*
*Group*	One	Introduction and Getting Started	2 h	Yes	No	n/a
*Group*	Two	Healthy Eating and Fats	2 h	Yes	No	n/a
*Group*	Three	Active Listening Skills	2.5 h	No	Yes	First practice of Active Listening Skills
*Group*	Four	Getting Active	2 h	Yes	Yes	Practice Opening and Closing of a call
*Group*	Five	Portion Control and Labels	2 h	Yes	Yes	Practice Active Listening Skills in content of call conversation
*Group*	Six	Motivational Interviewing	2.5 h	No	Yes	First practice call (duration 15 min), introduction of Motivational Interviewing skill set
*Group*	Seven	Maintaining Change	2 h	Yes	Yes	Full practice call (duration 20 min)
*One to One*	1^st^ practice Call	n/a	20 mins.	n/a	Yes	Assessment application of taught skill set
*One to One*	Additional practice calls	n/a	20 mins	n/a	Yes	Additional assessment if required

## Baseline screening assessment for eligibility (Table 6)

**Table 6:** Screening assessment

NDPS screening assessment - appointment 1
Ethnicity
Smoking status
Waist circumference [cm]
Height [cm]
Weight [kg]
Body mass index [BMI kg/m^2^]^a^
Body fat mass [kg]^a^
Visceral fat [kg]^a^
Body fat percentage^a^
Known first degree relative with T2DM
Known personal History of CHD
Known Gestational diabetes
Known previous result of IFG or IGT, prediabetes or Non-Diabetes Hyperglycaemia
GP surgery address
Participant’s address
Recruitment route
Screening and intervention preferred site
Have they been contacted about the national diabetes prevention programme or equivalent:define
Has the participant been admitted to hospital since their last visit, which has not already been reported and recorded for Serious Adverse Events and Adverse Events [SAE & AE] purposes

^a^measured using a Tanita body fat Bio-impedance composition analyser. The Tanita provides an electronic measurement of the body’s composition [tissue and fluid]. [TANITA - Hoogoorddreef, 1101 BE, Amsterdam, The Netherlands. Model BC-420 MA]

Following written informed consent, the screening assessment records the following information to the secure electronic trial database [eCRF] and on the Case Report Form [CRF].

### Outcomes and participant timeline

**Fig. 1:**
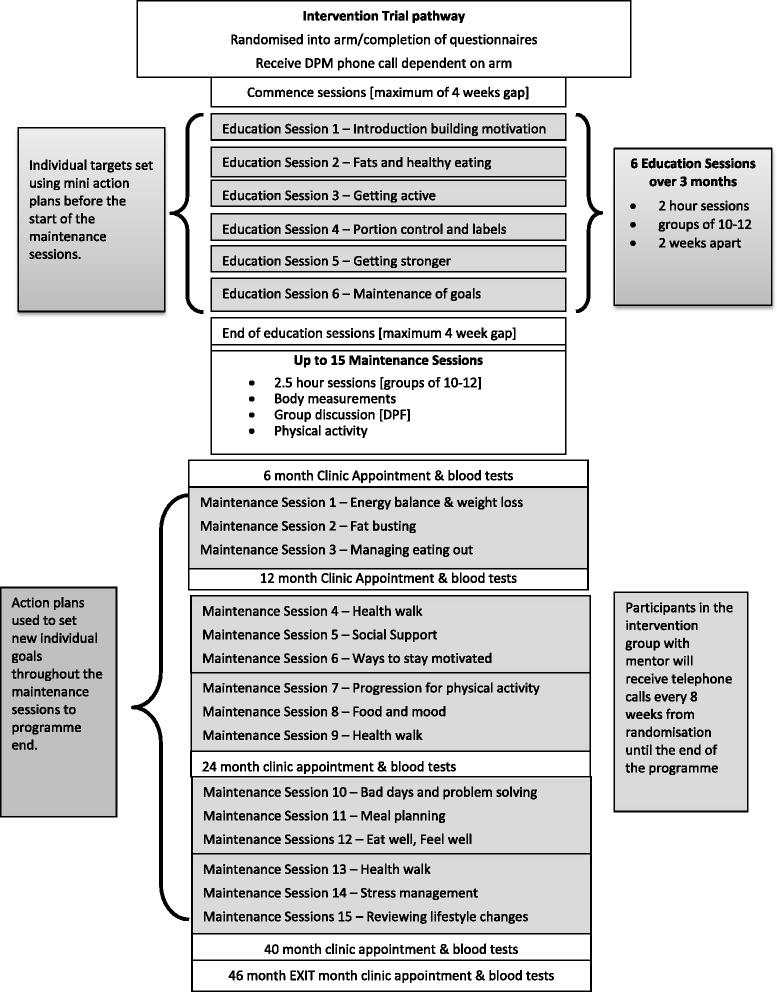
Schematic diagram schedule of registration, screening assessments, interventions, and time point assessments for Project 2 and 4

Tables 6, 7 and 8 and Fig. 1 outline the schedule of registration, screening assessments, interventions, and time point assessments. In the event of a raised fasting plasma glucose [>5.6 mmol/l] and/or HbA1c [≥42 mmol/l] in Project 1, the participant is invited back for repeat testing for fasting plasma glcuose and HbA1c. Classification of glycaemic categories and eligibility for randomisation is made on the basis of concordant paired baseline measurements, and sampling is repeated if necessary until paired concordant data allow classification. Samples for fasting lipids and insulin are also taken at this second appointment to form the baseline trial results. The lifestyle intervention comprises of 6 × 2 h education sessions of varying content for the first 12 weeks of the intervention which provide information and encourage decision making about behaviour change, increase motivation to change support lifestyle changes in relation to physical activity and diet with individual goal setting, action planning and self-monitoring. This core programme is followed by up to 15 maintenance sessions held 8 weeks apart from month 4 until intervention end which include facilitated discussion and physical activity circuits. Participants attend group based sessions, led by a trained DPF. On average there are eight participants in each group, with a maximum of 12. The groups remain together for the duration of the study, providing motivation for one another and a supportive network as the group bonds. All participants in the trial arms are expected to attend a 34 month active intervention, comprising of the 3 months education sessions, one month break post education and 30 months of maintenance sessions. All participants, including participants in the control arm, are required to attend clinic time point appointments at 6, 12, 24, 36, 40 and 46 month. Assessment includes recording to the secure electronic trial database and the time point CRF.

**Table 7:** Schematic diagram schedule of registration, screening assessments, interventions, and time point assessments for Project 2, 4 and 6^a^ for intervention and control groups

	*Screening*		*Randomisation*	*Post-randomisation*						*Exit*
	1^st^	2^nd^								
Timepoint [months]				4	6	12	24	36	40	46
Registration	x									
Eligibility screen	x	x	x							
Informed consent	x		x							
Fasting plasma glucose	x	x			x	x	x	x	x	x
HbA1c	x	x			x	x	x	x	x	x
Fasting lipid profile		x			x	x	x	x	x	x
Fasting plasma insulin		x			x	x	x	x	x	x
HOMA IR		x			x	x	x	x	x	x
Blood pressure		x			x	x	x	x	x	x
Height	x									
Weight	x		x	x	x	x	x	x	x	x
BMI	x		x	x	x	x	x	x	x	x
Waist circumference	x		x	x	x	x	x	x	x	x
Body fat mass	x		x	x	x	x	x	x	x	x
Body fat %	x		x	x	x	x	x	x	x	x
Accelerometer^b^			x	x	x	x	x	x	x	x

^a^Participants consented to Project 6 do not receive lipid and insulin analysis at baseline or at time points. These participants receive HbA1c and fasting plasma analysis at the 6, 12, 24 and 40 time points only

^b^Actigraph, LLC 17N, Tarragona Street, Pensacola, FL 32502, USA

**Table 8:**
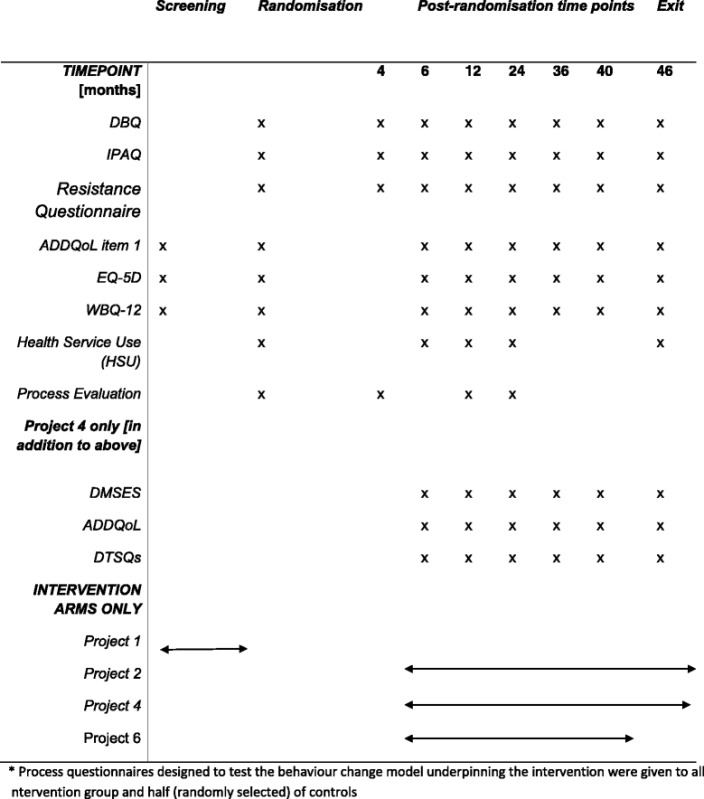
Schematic diagram schedule of questionnaire assessments by time point for Projects 2,4 and 6

### NDPS intervention (Additional files 1 and 2)

Participants with a randomisable category (Table 1) attend group based sessions, and intervention arms are not mixed in session attendance. Facilitators focus on group dynamics and the inclusion of all participants in discussions is encouraged. Maintenance sessions begin by reviewing behaviour change progress, followed by engagement in a physical activity circuit. An interactive topical discussion is included to aid continued learning and development and increase motivation. Each session is concluded by the inclusion of goal setting activity including action and coping planning, links are made from the solutions, and barriers discussed during the progress review to aid the setting of new goals. There is a substantial programme infrastructure for process analysis, intervention fidelity and participant adherence, and the intervention was developed in line with the MRC framework for the development and evaluation of Randomised Controlled Trials for complex health interventions [19].

This involved the following stages: 1) Extensive stakeholder involvement and needs assessment (interviews and discussions with service users [20] and potential service providers as well as with experts in the fields of diabetes, diabetes prevention, behaviour change and intervention delivery) 2). Reviewing of existing literature [3, 21–23] 3) Development (by the NDPS intervention development working group of an underpinning theoretical model describing the processes of behaviour change targeted by the intervention (Fig. 1) 4) Selection, based on evidence (where available) and expert opinion, of intervention strategies to deliver each of the intervention processes defined by the logic model.

#### Underpinning theory

Following consideration of several alternatives, the theory selected for developing the Intervention was the Process Model of Lifestyle Behaviour Change [24, 25]. This theoretical model was adapted from the Health Action Process Approach, [26, 27] and includes modifications to place a greater emphasis on self-monitoring, social support, use of coping plans and relapse management. The key intervention processes are i) increasing motivation (perceived importance of healthy lifestyle, self-efficacy for achieving healthy lifestyle, perceived risk and outcome expectations); ii) making a specific action plan (including plans for social support and for overcoming barriers (coping plans)) and iii) supporting maintenance through repeated ‘self-regulatory cycles’ of planning, self-monitoring and other feedback (for example on blood sugar), problem-solving to manage setbacks and revision of action plans. There is also an emphasis on empowering participants to develop autonomous motivation and to “make changes you can live with” to ensure that plans for lifestyle change are sustainable.

#### Structure

**Fig. 2:**
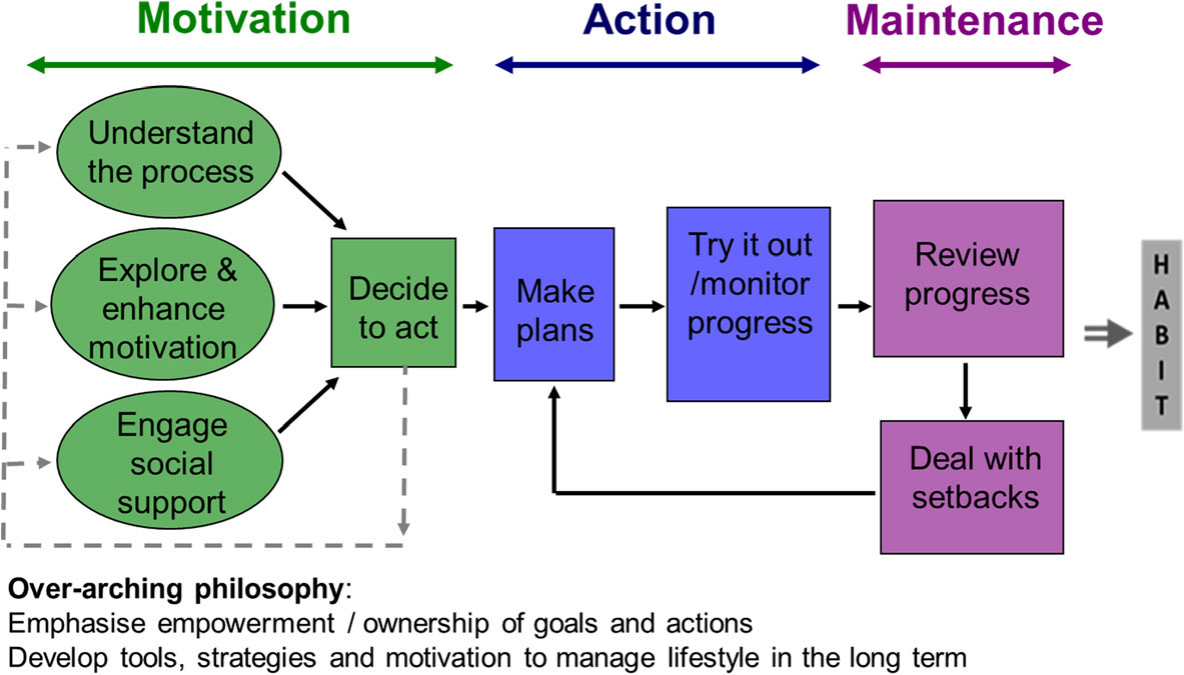
The Process Model of Lifestyle Behaviour Change [24]

The NDPS Lifestyle Intervention begins with six 2-h group based education/behaviour change intervention sessions spread over 12 weeks, After a 4 week gap, this is followed by up to 15 2.5-h group based maintenance sessions (including behaviour maintenance techniques and 50 min of structured exercise) delivered every 8 weeks (see Fig. 2 for session timings). The ingroup size is initially 10–12 participants, with options to merge groups over time (at the maintenance stage) if attendance diminishes. The total contact time (assuming all maintenance sessions are attended) is therefore 49.5 h, including 12.5 h of structured physical activity.

#### Content

A detailed description of the intervention is provided in Additional files 1 and 2. Briefly, the intervention aims to reduce diabetes progression through increased physical activity, specific changes in diet and weight loss. Behaviour change goals were set by participants but the NDPS intervention encouraged participants to shape these goals around 4 set targets; 1) If a BMI is over 30 kg/m^2^ then to work towards a 7% weight loss in the first 6 months and maintain until study end, 2) Reduce the amount of fat and specifically saturated fat from diet, 3) Work up to 150 min of moderate intensity physical activity on 5 days of the week or more which can be achieved through an increased step count and 4) work up to increasing muscle strength activity to 160–300 reps on 2–3 days of the week as these are known to induce clinically meaningful changes in diabetes risk for people with hyperglycaemia [3, 4].

Behaviour change techniques are selected to facilitate each of the processes identified in the behaviour change model and delivery strategies and detailed materials are specified for each change technique. The programme delivers these strategies and techniques in a logical sequence (Fig. 2; Additional file 1). This includes a focus on one type of behaviour (diet or physical activity) in each of the first six sessions and a combined focus in the maintenance sessions. In the first six sessions, activities to implement behaviour change techniques are combined with activities to build understanding around what lifestyle changes to make (e.g. what is a healthy diet?, what is moderate intensity activity?) and how to change them (e.g., how to make a SMART-ER action plans, how to identify and solve problems). Maintenance sessions then focus on reviewing progress, problem-solving any setbacks and reviewing action plans, as well as delivering a 50-min session of supervised, structured physical activity. In terms of specific strategies for lifestyle change, we present national guidance on healthy eating (based on the EatWell Guide [28] and compared existing diet to these recommendations. However, participants were free to decide what specific changes to make. We encourag participants to choose physical activity that they can build into their existing lifestyles and present ideas for muscle-strengthening exercises. Structured activity is presented in the maintenance sessions as a) as an opportunity to practice techniques in a safe, supervised environment, and b) to reinforce the perception the programme is serious about encouraging physical activity as well as dietary change, as people may tend to focus more on the dietary aspect of such programmes [29] To promote sustainability of lifestyle change, we advise participants to make a series of small, achievable changes, rather than dramatic changes. We encourage participants to prioritise ideas for change that would not detract from their enjoyment of food (for dietary changes) or that would be enjoyable or easy to build into a routine (for physical activity [30]).

#### Delivery and facilitator training

The initial six session “core” intervention is delivered to each group of participants by a single facilitator (DPF) where possible. The sessions are semi-structured with a printed session plan and a set of Powerpoint slides for each session. Presentation of the session is based on the slides, with a mixture of didactic presentation (providing information) and interactive activities (slides are paused and participants engage in a discussion or other interactive activity, such as problem-solving, or action-planning). Content is tailored to build on the existing knowledge and skills of participants. The DPF spends time with each individual who needs help during action-planning activities and tries where possible (within the limits of group-based delivery) to elicit and respond to individual motivations, barriers to change or other concerns. Flipcharts are used to keep track of ideas from the group in a number of interactive activities and additional printed information materials (handouts) are available for a number of sessions covering a range of topics (for example, healthy eating, why weight loss plateaus and what to do about it, a pictorial diagram of a set of muscle-strengthening exercises that can be performed at home/without any equipment),.

*NDPS diabetes prevention facilitators (DPF)* are recruited from varied backgrounds and experience, including academic qualifications in nutrition or physical activity, fitness industry, and physiotherapy. A 5 day training course was developed and delivered (primarily NM and CG). The course content focuses on developing behaviour change skills (Additional file 2), updating knowledge (for example on healthy eating), using the intervention materials, delivering the session content using the slides and session plans and data collection issues (including providing data for the research study). The style of delivery is important and we trained the facilitators to use person-centred counselling techniques based on motivational interviewing (open questioning, affirmation, reflective listening, summaries, use of the Ask-Tell-Discuss technique for information exchange) [31, 32] to promote autonomous motivation and to deliver all of the intervention content. A selection of 3–4 audio recordings per facilitator of session delivery is reviewed to check fidelity of intervention delivery. Facilitators also self-complete a fidelity checklist at the end of each session. The senior staff use this data to a) develop training updates and b) give the facilitators individual formative feedback. Supervision meetings are held approximately every 2 months, where barriers and solutions to delivery are discussed. The intervention is delivered in local community venues (e.g. community halls, University premises etc.).

## Control group(s)

Participants in the control groups (Projects 2 and 6) are offered attendance at the first available planned session for their chosen intervention location. This session is offered within 4 weeks of being randomised into the control arm of the programme. If a particpant was unable to attend this session the next planned session was offered to them. The 2-h control session offers the same education as the intervention but in condensed form. The content relates to ‘pre-diabetes’ and T2DM risk and weight, physical activity and dietary behaviour in relation to T2DM risk and amangement Control participants are asked to attend follow up appointments at 6, 12, 24, 36, 40 and 46 months and to complete questionnaires. Process questionnaires designed to test the behaviour change model underpinning the intervention were given to all intervention group and half (randomly selected) of controls. In the areas where the study took place, the control session matched closely what is offered in primary care as a standard pathway of care, to those newly diagnosed with a ‘prediabetes’ category (Table 1). Participants with T2DM in control and intervention groups receive standard care through their normal GP diabetes service in line with local primary care diabetes management guidelines.

## Process evaluation (Table 9)

**Table 9:** Process Evaluation questionnaire measures

*Process*	*Measures [all at 0, 4, 12, 24 and 40 months]*
*Understanding the process of behaviour change*	Brief questionnaire piloted/refined with feedback from 15 people and (along with all the newly developed/adapted measures below) validated in a separate study [66]: Covers knowledge about how to make permanent changes to behaviour, how to get and stay motivated, the perceived importance of social support, knowing how to overcome barriers and having skills to manage food cravings (the key processes underpinning the NDPS intervention model).
*Explore and enhance motivation [Perceived Importance of lifestyle change]*	Perceived importance of eating a healthy diet [with a brief definition provided]: We used a 0 to 10 visual analog scale [VAS] and have also adapted the Intrinsic Motivation Inventory [67, 68] by reducing the number of items to 4 and providing 3 specific intrinsic motivations that should be relevant to the target group and/or which are targeted by the intervention [helping to control my weight; reducing my risk of getting heart disease; contributing to my sense of well-being]. Perceived importance of doing at least 150 min/week of moderate to vigorous physical activity [MVPA]: We also used a 0 to 10 VAS and the same 4 items from the IMI.
*Explore and enhance motivation [Confidence about ability to change]*	Self-efficacy for healthy eating: we used a 5-item reduced/modified version of the Weight Efficacy Life-Style Questionnaire [69]. We also used a 0 to 10 VAS scale to assess confidence about eating a healthy diet [definition provided] over a] the next month and b] the next 12 months Self-efficacy for achieving a healthy level of Physical Activity: we use a 5-item physical activity self-efficacy scale [70]. We also used a 0 to 10 VAS scale to assess confidence about being able to achieve a healthy level of physical activity [definition provided] over a] the next month and b] the next 12 months.
*Identify and engage sources of social support*	Social support for healthy diet: we used a 5-item adaptation of the Sallis et al. scale developed by Norman et al. [71, 72] Social support for healthy level physical activity : we used a 5-item adaptation of the Sallis scale developed by Roesch et al. [71, 73]
*Intention Formation*	Intention for healthy diet: We developed a brief 4 item measure, using a 5-point Likert scale to assess the level of agreement/disagreement with statements about intention to a) eat healthily and b) adhere to the three main healthy eating recommendations of the programme. Intention for physical activity: We developed a 3 item measure using 2 items from Sniehotta et al. [74] and a further item about doing moderate intensity activity on 5 days of the week. We used the same 5-point Likert response scale as above.
*Action planning*	Level of engagement with action planning process. 1. From coding of completed action plans (intervention group only) to indicate level of engagement with the key elements of goal-setting, coping planning and social support planning, as well as participant ratings of how useful the plans/reviewing of plans were. 2. We used 4 items on action planning and 3 items on coping planning from the scale developed by Sniehotta et al. [75]
*Self-Regulation (monitoring and problem-solving relating to diet and physical activity)*	Frequency of self-monitoring and relapse management activities: We used two pre-existing items on self-monitoring [74], two newly constructed items on attempts to identify and solve problems, and one (new) item on general salience of physical activity aims, all over the last month. Frequency of weighing: 1 item from Linde et al. [76] Barriers/problem-solving (intervention group only): We coded progress-review sheets and coping plans used in the intervention under four sub-headings: Practical barriers; People and places; Thoughts and feelings; Other. Level of engagement in this problem-solving activity was defined in terms of the number of items recorded on the written plans in a defined time period prior to measurement.
*Empathy/empowerment*	Client Satisfaction (intervention group only): Items from the Learning Climate Questionnaire [77] to assess how much empathy/empowerment and engagement participants felt they have with the intervention facilitators.
*Quality of intra-group interactions*	Physical Activity Group Environment Questionnaire (intervention group only): We selected the 6 items with most face validity for this intervention [78]
*Affective response/reinforcement (enjoyment of or other positive reactions to the target lifestyle changes)* *Managing impulsive processes (for unhealthy eating)* *Body Image Dissatisfaction*	Affective response to physical activity: we selected four items from an eight-item version of a Physical Activity Enjoyment Scale (PACES; [68]) Items were selected to represent conceptual diversity [several items in the original scale simply use different words for ‘enjoy’].. Affective evaluation of eating a healthy diet: We selected two items from the Interest/Enjoyment scale of the Intrinsic Motivation Inventory [67] asking about enjoyment of “healthy foods” [with a definition provided] and added an item asking for level of agreement with the statement “I have found a diet that is both healthy and enjoyable”. We selected ten items from the 18-item version of the Three Factor Eating Questionnaire [79] These items represent the sub-scales for ‘cognitive restraint’ [all six items] and ‘uncontrolled eating’ [four of nine items], Perceived Body Image: We used an existing two-items Perceived Body Image questionnaire [79, 80].

Process evaluation is an integral part of the trial and gives greater explanatory power to the outcome measures a better understanding of the mechanisms of action, likely generalisability of the intervention, and assesses intervention fidelity and participant adherence. The process evaluation includes:

- Higher level process analysis: Tests a physiological causal model for diabetes prevention, and examines the mediating role of changes in dietary and physical activity behaviours on the intervention effect on T2DM incidence. This analysis uses the T2DM incidence, accelerometer data, and short form IPAQ questionnaire [33] (to measure physical activity) and the Dietary Behaviour Questionnaire (DBQ) adapted from the original Fat and Fibre Related Diet Behaviour Questionnaire [34] to measure dietary behaviour change
- Intermediate level process analysis: Examines the mediating effect of intervention exposure on behaviour change and other study outcomes, using individual level measures of intervention attendance on study outcomes. Fidelity of the intervention is assessed in two ways. Firstly, by scoring of delivery quality, based on a checklist completed by DPF to provide an overall score relating to style of delivery and content provision. The BECCI [35] and MITI coding systems [36] are examples of this approach. Secondly, by coding of recordings of delivered sessions by independent raters (in a subsample).
- Finer-grained process analysis: Tests the underpinning theoretical model of behaviour change, and whether the intervention leads to changes in targeted processes such as understanding the behaviour change process, and motivational variables such as perceived importance and self-efficacy, and social support. The mediating effect of changes in process variables on changes in dietary and physical activity behaviour and weight are examined using the process measures described in Table 9.

### Primary outcomes

The primary end point in **Project 2** is the diagnosis of T2DM based on paired fasting plasma glucose results both ≥7.0 mmol/l, or both HbA1c results ≥48 mmol/mol at 46 months after enrollment. One time point result in this range [≥7.0 mmol/l, or ≥48 mmol/mol] triggers a repeat fasting sample for fasting plasma glucose and HbA1c to confirm a diagnostic end point, and a T2DM primary end point is only confirmed when both samples are concordant. The primary end point in **Project 4** is HbA1c at 46 month project end as a measure of glycaemic control between groups with screen detected T2DM. Power estimates and sample size are based on biologically relevant differences in mean HbA1c between groups at project end. The primary end point in **Project 6** is HbA1c at 40 month project end as a measure of glycaemic control between groups.

### Secondary outcomes

In Project 2, 4 and 6 there are six sets of prespecified secondary end points in each trial with analysis between arms for these outcomes at trial end, and at interval time points, between groups. These are a] homeostasis model assessment [HOMA] estimates of insulin sensitivity and beta cell function based on fasting plasma glucose and insulin levels [37], b] exercise levels based on accelerometer data and self-reported activity and International Physical Activity Questionnaires [IPAQ] [33], c] dietary intake based on self-reported Diet Behaviour Questionnaire designed in house [DBQ] d] weight, body fat mass, visceral fat, BMI and waist circumference e] Changes in the questionnaire designed to measure health status [EQ–5D], Well-Being Questionnaire, 12 items [WBQ-12], item 1 from the Audit of Diabetes-Dependent Quality-of-Life questionnaire [ADDQoL], [38, 39] and study specific questionnaire data f] weighted composite lifestyle change score based on changes in weight, BMI, waist circumference and exercise levels. One pre specified secondary analysis in Project 2 is to analyse the cumulative incidence of T2DM in a composite analysis group of the two intervention arms combined, compared to controls. This will be undertaken if the primary comparison between intervention groups in Project 2 end is not significant. In Project 4 in addition to these secondary end points we also collect diabetes specific questionnaire data on Diabetes Treatment Satisfaction Questionnaire [DTSQs] and Diabetes Management Self-Efficacy Scale [DMSES] at each time point [40, 41]. In Project 3 the primary objective is to recruit, train and retain volunteers with T2DM to be DPMs. However, as secondary end points we also collect DPM biometrics at, 0,6, 12,24 and 36 months [HbA1c, weight, blood pressure, waist circumference, body fat measurements and BMI], and at the same time points collect changes in exercise levels [based on self-reported activity and IPAQ questionnaire], changes in dietary intake based on self-reported DBQ questionnaire, quality of life and psychological well-being measured by WBQ-12, DMSES, ADDQoL and DTSQ as individuals who provide social support through volunteering experience less depression, heightened self-esteem and self-efficacy, and improved quality of life, and improved health outcomes. We also collect data on the DPM perceptions of their capacity to deliver the NDPS intervention measured by focus groups and confidential views on DPM intervention efficacy in a study specific questionnaire and focus groups. These secondary DPM data will be analysed longitudinally within group between baseline and end point.

## Sample size estimates, original and revised power calculations

Original NDPS sample size and power estimates [2010] were based on transition rates from IFG [>6.1–<7.0 mmol/l] to T2DM in Northern European white populations. In the Danish ADDITION study [42] annual transition rates were 11.8% in 1821 individuals with IFG [mean age 60 years; mean BMI 29.1 kg/m^2^ and similar to our population] with a transition rate highest in the first year. In the Inter99 study [43], the transition rate was 10.4% for those with IFG – IGT combined in younger and slimmer participants [age 42.7 years; BMI 27.7 kg/m2] although 50% had already had diet and lifestyle interventions. We assumed an annual incidence rate of 8% was reasonable, at a 22% cumulative incidence over 3 years [allowing for incident cases]. Meta- analysis of published diabetes prevention trials at the time [44] suggested an estimated effect size effect sizes based on a meta - analysis effect size of 0.51 [95% CI 0.44–0.66] of diet and lifestyle interventions in IGT suggesting we could look for an equivalent effect size in our intervention groups with a 4% annual progression in the intervention group [12% cumulative incidence over 3 years allowing for incident cases]. A 36 month intervention asymmetrically randomized controlled trial of 170 controls [standard care] and 390 intervention participants gave 80% power at 5% significance level to detect this difference in proportion progressing [project 2]. We hypothesized that the intervention effect size would be further enhanced by additional support from the DPM with enhanced weight loss, weight loss maintenance and exercise adherence, which was poor at DPP end [3]. The lowest quartile dietary fat intake participants in the Finnish Diabetes Prevention study [45] had half the T2DM incidence compared to highest quartile, with an overall mean 58% reduction, and we hypothesized that the enhanced DPM group would experience a further reduction in the T2DM incidence rate to 2% per annum [6% cumulative incidence over 3 years], then 390 additional IFG participants will provide 80% power at 5% significance level to detect this difference in proportions progressing compared to the intervention alone.

The move to a diagnostic cut point of HbA1c ≥48 mmol/mol for diabetes [13, 14] largely supplanted diagnosis based on fasting glucose or oral glucose tolerance test criteria. This policy generates a large population with NDH HbA1c ≥42–<48 mmol/mol iand UK policy guidance [15] is that these patients should receive intensive lifestyle advice and annual HbA1c monitoring. This change occurred during the early accrual period of NDPS (largely based on glucose entry criteria at inception) and we adapted the programme to take this into account as the HbA1c defined population is likely to become the dominant ‘pre diabetes’ population in UK practice in the next few years. Meta - analysis of prediabetes transition rates [46] suggest that this HbA1c defined NDH population have an annualised T2DM incidence of 4.7% [95% CI 3.7–5.9] for a glucose based T2DM end point, independent of any concurrent fasting glucose classification. In earlier meta analyses, annual progression rate to T2DM based on a NDH, independent of any concurrent fasting plasma glucose data, was 5–10% per annum, with an estimated annualised transition to T2DM of 7.5% at a mean HbA1c of approximately 44 mmol/mol [47]. In NDPS, mean HbA1c in the NDH group was 43.7 [SD 1.4] mmol/mol for the earlier screened population [Project 1]. Lipska et al. [48], demonstrated that the combination of both NDH range HbA1c and IFG was associated with a relative risk of T2DM of 26.2 [95% CI 16.3–42.1] compared to those with neither, with a transition rate to T2DM of 7% per annum in the combined group. Similar risk signals at this level are apparent in other populations for a combined lower range IFG (>5.6–<6.1 mmol/l) and NDH with transition rates approaching 10% per annum, at an age and gender adjusted hazard ratio of 38.6 [95% CI 27.6–54.0] compared to normal glycaemic status [47, 48]. Because of this policy change [13–15] and these data, since 2014 we have also randomized participants into Project 2 if they had an HbA1c ≥42 to <48 mmol/moll combined with an elevated fasting plasma glucose ≥5. 6–<6.1 mmol/l Current revised power estimates reviewed and approved by independent DMEC, TSC, sponsor and funding body are based on a final accrued sample size of 972 in Project 2, with a 46 month follow up and end of trial 1.4.18. Current retention and follow up data, and above assumed transition rates as above, gives 99.7% power to detect these difference between controls and intervention, 84% power between controls and intervention plus DPM arm, and 78.6–80.2% power to detect a difference between the two intervention arms. In Project 4 in order to have 80% power to detect a difference of 0.5% in HbA1c between groups at end of trial, (0.4 standard deviations based on screen detected HbA1c distribution) requires 99 participants per group, and to allow for a drop-out rate of 20%, a total of 375 individuals will be randomised. The transition rate to T2DM for subjects with NDH and a normal fasting glucose (<5.6 mmol/l) is not clear, and Project 6 is not powered to detect differences in transition rates to T2DM, but is an observational RCT in this understudied group.

## Statistical analysis plan

For project 2 we will use an intention-to-treat approach. For binary outcomes we will use the chi-squared test or logistic regression if adjustment for baseline imbalances is required; for continuous outcomes we will use the *t*-test for comparison of two arms or analysis of covariance for comparison of all three. For time-to-event outcomes the proportional hazards model will be used with time measured from randomisation until the end of the study or drop-out [censoring] or diabetes is diagnosed. Secondary analyses will include a per-protocol analysis and analysis of secondary outcome measures. For determinants of progression we will use logistic regression and the proportion hazards model with the time of the onset of T2DM as the outcome measure in each treatment arm separately as well as compliance with the intervention. If necessary we will use multiple imputation to assess sensitivity to missing data. For project 2, the primary outcome measure is progression to T2DM by study exit. This will be analysed using a logistic regression model including a covariate to account for the different potential follow-up times at baseline. A secondary analysis will adjust for potential prognostic factors which will be agreed prior the final analysis. Secondary continuous outcome measures will be analysed using a general linear model in a similar fashion using the study exit. Time-until progression to T2DM will be analysed using a discrete time survival analysis model. A longitudinal analysis of the continuous secondary endpoints will also be undertaken to assess for differences over time using a random effect approach. Effect sizes will be estimated for all two-way comparison of intervention group and no adjustment for multiple testing will be undertaken. The primary analyses will be based on the intention-to-treat population but, if appropriate, a CACE analysis will also be undertaken for the primary outcome. Adverse events will be tabulate but no formal comparison will be undertaken. A similar approach will applied to Project 4 with HbA1c as the primary outcome between groups with T2DM. In Project 6 a similar approach will be applied with HbA1c as the primary outcome between groups.

## Recruitment strategies

### Methods: assignment of intervention and allocation

Randomisation of participants is conducted using a dedicated function in the trial data management system. The randomisation mechanism consists of a pre-prepared random list of codes [for the Intervention and Control groups] that are stored in a table in the trial database. To reduce the risk of predicting the next allocation while maintaining a reasonable even spread of intervention and control patients, the list is constructed of blocks of 17 codes 3 control, 7 intervention and 7 intervention + DPM to approximate the proportions of 170:390:390 respectively.

The list were built prior to the start of the programme using standard Microsoft tools. Randomisation is asymmetric to deliver sample sizes described. Projects 4 and 6 are randomised using the same method in the trial data mangement system. Project 4 are randomised to a 1:1:1 ratio and the Project 6 is randomised to a 1:1 ratio to control or intervention only.

### Methods: data management

All study data are recorded and stored on the study database [CRF/eCRF]. Each clinic appointment has a dedicated paper CRF designed for optimal collection of data in line with study protocol compliance and regulatory requirements. CRF’s are prepared in line with protocol development and version controlled. All clinical measurements are recorded on both the CRF and the eCRF simultaenously at the time of the clinic appointment. Data entry and daily data management is performed via the dedicated secure database website. The NDPS Data Management Plan describes the intended procedures for managing data in the NDPS. An updated report of missing/erroneous data is generated weekly to aid data cleansing in real time. Each participant has a data cleansing section on their database page which records all data checking activity. Questionnaire entry is monitored weekly via the NDPS update report, generated directly from the database. This allows weekly monitoring of registration, overall accrual for each project, accrual by site, withdrawal rates, the rate of registration versus consent to screening, and adherence to intervention sessions.

### Methods: biochemical analyses

Fasting plasma glucose [hexokinase/G-6-PDH method Architect c8000: Abbott Diagnostics, Maidenhead, UK], HbA1c [Affinity high performance liquid chromatography; Hb9210: Menarini Diagnostics Ltd., Wokingham, RG41 5RA, UK], Total Cholesterol: enzymatic: cholesterol oxidase/hydrogen peroxide, Triglyceride: Enzymatic: glycerol kinase/hydrogen peroxide, HDL cholesterol : Direct method using a specific detergent to solubilise HDL Cholesterol/cholesterol esterase; LDL cholesterol - Calculation using the Friedewald formula:LDL-C (mmol/L) = Total CHOL (mmol/L) - (HDL-C (mmol/l) + TRIG (mmol/l) Insulin - solid-phase, enzyme-labelled chemiluminescent sandwich immunometric assay (Siemens Healthcare Diagnostics, Camberley, GU16 8QD).

## Ethics and dissemination

### Ethics approval

NHS Research Ethics Committee [REC] - NRES Committee East of England Essex reviewed applications for research and gave a favourable ethical opinion for proposed participant involvement on the 3th January 2011 [REC number 10/H0301/55]. To date 40 substantial protocol amendments, and 38 minor amendments have been made to the programme along with 2 submissions for a Site Specific Information [SSI] to allow two further NHS secondary care trusts to conduct the research at their particular locality All significant protocol amendments have been REC reviewed and approved, and approved by host organisation and NIHR programme board. In line with Ethical approval for this programme, participants can withdraw from the programme at any point, without having to give reasons for withdrawal. Participants who do decide to withdraw are given the option to withdraw completely from all further contact, or to agreed to further contact for time point blood sampling for biochemical end points and diagnosis as per protocol, but without intervention. Participants reaching a prespecified end point of new T2DM diagnosis leave the programme and are managed by normal primary care services.

### Consent, security, and confidentiality

Participants are provided with the patient information sheet [PIS] for all NDPS trials prior to registration or at the time of registration. At registration verbal consent is obtained from each participant prior to recording details on the study database [eCRF]. The study is fully explained by a member of the team at registration and the partcipant is given the opportunity to ask questions prior to written informed consent being taken at the first screening clinic appointment. Written informed consent is taken from participants prior to both screening procedures being undertaken and prior to randomisation into any group. The multiple PIS and consent forms are summarised in the Additional files 1, 2, 3, 4 and 5. Data is stored in a secure database on the Clinical Trials Unit [CTU] Microsoft’s SQL Server system at the University of East Anglia. Access to the server is restricted absolutely to CTU data management staff and NDPS staff working on the programme. User access is authorized by the PI or CI and the database is protected by usernames and passwords. The server and its contents are covered by the UEA IT regulations and policies. Data on the server is backed up nightly and regularly archived to off-site storage. In addition, other ‘quick-access’ backups are taken by the NDPS Database Manager and held on-line to facilitate rapid recovery of recently changed data. Each user has an individual username and password without which they are able to access the system and any attempt to access pages ‘deeper’ into the NDPS data entry system will automatically be re-routed to the login page. Traffic between the user’s PC and the server is secured using standard SSL [Secure Sockets Layer] technology. Unattended PCs left logged onto the system are ‘timeout’ after a period. All identifiable data is stored in locked cabinets in authorised card entry offices or clinics. Access to the final trial dataset will be limited to CI, PI and programme statistician.

### Methods: monitoring

The NDPS has a dedicated independent Data Monitoring and Ethics Committee [DMEC]. The committee reviews accumulating data and advises the Chief and Principal Investigator directly and indirectly via the programme statistician, on the future management of the trial. The committee reviews the safety, efficacy, quality and compliance data of the programme at yearly committee meeting and bi-monthly reports. The DMEC is privy via a closed session with the core statistical team, to interim comparisons by arm and may determine the form of data analysis, end point adjudication and interim analyses to be undertaken. The DMEC report is presented to the Trial Steering Committee [TSC] which meets 6 monthly. The DMEC and TSC terms of reference and membership are available. The study is fully GCP compliant and is audited by the host organization Research and Innovation Department. The programme receives formal quality assurance support from the Norwich CTU including the documentation of all quality procedures in a Quality Management Plan and assistance to set up the TSC and DMEC with roles and responsibilities of these committees documented in line with established guidelines.

### Protocol amendments

All protocol amendments have been made in accordance with the Research Ethics Committees [REC] standard operating policies. All amendments have been submitted for REC, Research Health Authority [RHA], sponsor Research & Innovation Department approval prior to implementing. All significant amendments to protocol have been reviewed by funding body [NIHR PGfAR]. All amendments have been documented in a protocol amendment tracker [see Additional file 3]. The initial protocol was approved by REC in January 2011, and current protocol is version 13]. Protocols v2 - v4, v7, v9, v10, and v12 were for minor textual changes in materials. v5 allowed new geographical areas to be accessed to maintain accrual, v6, v8 and v9 recorded additional supplementary studies, including epigenetic analysis [V6], iron balance studies [v9], and Project 6 to take into account new categories of NDH [v8]. v11 [3.4.14] was a larger scale amendment that allowed access to new populations in Suffolk and Essex to maintain power, to prolong follow up to 46 months and revised inclusion criteria for randomisation into Project 2 that recognised the recent development of new diagnostic criteria for diabetes and NDH based on HbA1c criteria.

### Ancillary and post - trial care

Participants with screen detected T2DM randomised to Project 4 also receive standard best practice diabetes care through their general practice. The programme does not interfere with any element of their normal diabetes care. All participants have access to a DPF, the Principal Investigator or Chief Investigator if they wish to discuss their diagnosis and implications of these results. However, in all cases the participants are encouraged to discuss their diagnosis/results with their GP. Post - trial (April 2018) all participants in this programme will be maintained in primary care follow up in line with normal clinical best practice at that time (2018). All participants and GPs will be written to with a data summary, diagnostic categorisation, and suggested further follow and care plans.

## Dissemination

The main NDPS trial(s) will be submitted for publication in July 2018. All participants and participating GPs will be sent a summary of trial outcomes, linked to current models of care and diabetes prevention strategies at the time.

## Economic evaluation

We will estimate the cost-effectiveness of the Norfolk DPS intervention [with and without DPM], compared to the control arm, for individuals identified as having IFG or T2DM. The level of resource use associated with the intervention [including that associated with the education and maintenance sessions, as well as DPM input] will be estimated along with other NHS resources that are potentially related to the intervention [e.g. referrals to dietician and GP visits]. Appropriate unit costs e.g. [49] will be assigned to all resource items. The economic evaluation will use the EQ-5D [50] as the main measure of effectiveness, enabling QALY [quality adjusted life year] scores to be estimated. Costs and QALYs incurred in future years will be discounted. Subsequently, the incremental cost-effectiveness ratio will be calculated and compared to a range of cost-effectiveness thresholds [51]. In order to characterise the level of uncertainty the cost-effectiveness acceptability curve [52] will also be presented. Sensitivity analysis will also be undertaken to assess the robustness of conclusions to key assumptions that are made within the economics analysisIf there are differences in T2DM incidence or in cardiovascular risk factors at the end of project 2, the ongoing effects, health care costs and cost effectiveness of the intervention will be modelled over the projected lifetimes of participants. Risks of complications of T2DM will be based primarily on the UKPDS risk engine [53, 54], risks of coronary heart disease and stroke will be based primarily on QRISK2-2016 [55]. NHS costs of T2DM, cardiovascular risk factors and diabetes complications will be based on published estimates. Residual death rates will be based on UK Office of National Statistics estimates. This will be a multistate life table model, in which, for each surviving person each year, values of risk factors, adverse outcomes will be simulated, based on the values in the preceding year and assumed annual changes in risk factors and on incidence of adverse outcomes. The quality of life of each individual each year will be calculated, based on the presence of adverse outcomes, using primary quality of life data from project 2, and published estimates of QALYs associate with the respective outcomes. Simulation of the cohort will end when 99% have been simulated to die. As with the within-trial economic evaluation, incremental cost effectiveness ratios will be estimated and cost effectiveness acceptability curves will be presented. A probabilistic sensitivity analysis will be conducted, incorporating parameter and stochastic uncertainty. Other sensitivity analyses will explore the sensitivity of the primary analysis to assumptions about key parameters such as risks of T2DM, coronary heart disease, stroke, death, quality of life, costs and discount rates.

## Discussion

The need for effective diabetes prevention strategies to be developed at scale in existing health care systems was recognised by the announcement of the NHS England diabetes prevention programme in 2015 [9, 56]. This national programme was largely predicated on existing evidence of diabetes prevention benefit in very large well - resourced clinical trials [3–5] in highest risk individuals. There is a substantial translational literature, examining the more variable impact of shorter term and less intense lifestyle interventions on glycaemic and metabolic outcomes in high risk groups [6–8]. Recent meta - analysis [8] of 44 clinical studies (8995 participants) has shown that modified, simpler and shorter interventions based on the US diabetes prevention programme (DPP), and using different delivery models, is associated with significant weight loss and reductions in HbA1c, but the diabetes prevention impact of these models remains uncertain. There is also still uncertainty over the best approach to find those at highest risk of T2DM for a clinical or research intervention, and over which glycaemic category should receive the intervention [6]. It seems sensible to develop and test research interventions that would be translatable to normal clinical practice, that recognises the complexities of risk categorization, and the need to reduce intervention costs. The NDPS will address many of these issues and reports in 2018.

The arguments underlying the NDPS programme (2011–2018) are similar to those used later in the NHS England prevention programme (2015), that the costs of research based diabetes prevention interventions are prohibitive, that new approaches are needed to deliver the intervention (based on group work), potentially using lay mentors to deliver the intervention and recognizing the need to limit impact on pressured primary care teams. The value of the NDPS programme has increased since inception, as it will answer many of the current questions about delivery of the NHS England programme [9, 56], and also provide crucial trial outcomes trial data for the high risk group with non - diabetic hyperglycaemia (NDH). The trial evidence in this population is relatively limited [8, 9, 56] and the shift in diagnostic criteria for diabetes [13–15] from glucose based to HbA1c based diagnostic criteria has created large populations with NDH. There are also limited data on the diabetes prevention intervention benefit in the population with IFG, based on a fasting plasma glucose test, rather than impaired glucose tolerance based on a time consuming (for patients and staff) oral glucose tolerance test, which would not be widely used for mass population screening for those at high risk in a non - research active primary care setting [16, 17]. These at risk glycaemic categories (based on HbA1c and fasting plasma glucose) have been for some time the largest at risk glycaemic populations identified in UK general practice. The NDPS programme recognises the complexity of the these various categories of ‘prediabetes’, and randomizes clinicallt recognizable combinations combinations of these categories into trial(s) and will provide evidence for progression and risk reduction in these categories.

One important element of NDPS is that screening programs or case finding in general practice for people at highest risk with IFG, IGT or NDH will also generate significant populations with screen detected T2DM. In NDPS we randomize these participants into the same intervention groups (Project 4). This trial will answer the question of whether a diabetes prevention intervention also has glycaemic outcomes benefit in people with screen detected T2DM, and replicates normal clinical practice.

Peer support can be defined as support from persons who have the same health condition as the people they assist and who experience similar challenges of those with the condition [10–12]. The use of lay mentors or community health care workers to support a lifestyle intervention, and reduce the very high costs of more intensive research interventions was recognized by the early triallists [3]. Evaluation of the role of lay supporters generally report positive participant perceptions and biomedical outcomes [10–12]. Although the role is variously described most of the diabetes literature has examined impact on the clinical care of people living with diabetes [10–12] and the higher the intensity of the program the greater the reduction in HbA1c [12]. The use of unpaid volunteers with T2DM themselves to provide peer support in the context of T2DM prevention trial is a novel approach [57]. This model offers real opportunities to devlop a new volunteer workforce with T2DM who have a shared interest in diet, lifestyle and glycaemic outcomes to the participants they are supporting in a diabetes prevention intervention In NDPS, the diabetes prevention mentors deliver support by telephone, and telephone communication has been found to be acceptable by patients and is commonly a preferred and convenient option [58, 59].

The screening element of NDPS [project 1] uses existing GP practice databases to identify those at highest risk for further screening, with minimal impact on GP workload, using standard and very simple database queries in the commonest UK database systems, where search terms are available for almost all patients. This deliberate approach was developed to limit workload within practices and encourage practice engagement without using modelling from more complex risk prediction tools where data on predictive variables may be unavailable [18, 60, 61]. The data from the screening element of this programme will have value in itself, as it will allow estimates of the population size of the various glycaemic categories in normal GP practice databases. The simple search criteria used in NDPS were also chosen to ensure NDPS was concordant with the 2010 NHS national vascular screening programme [62, 63]. This mass primary care cardiovascular risk screening programme in England uses age >40 year and BMI >30 kg/m^2^ as criteria for further fasting plasma glucose or HbA1c testing, is now a mandatory requirement for local authorities in England [63].

The NDPS programme is projected to reach full sample size and power on current accrual rates and will report in mid – 2018 for all trials.

## Additional files

Additional file 1: Description of intervention. (DOCX 252 kb)
Additional file 2: DPF Training manual. (DOCX 309 kb)
Additional file 3: Protocol version tracker. (DOCX 24 kb)
Additional file 4: SPIRIT checklist. (DOCX 33 kb)
Additional file 5: TIDieR checklist. (DOC 120 kb)

## Abbreviations

BMI: Body mass index
CCG: Clinical commissioning group
CTU: Clinical trials unit
DMEC: Data monitoring and ethics committee
DPF: Diabetes prevention facilitators
DPM: Diabetes prevention mentors
GP: General practitioner/GP surgery
IFG: Impaired fasting glucose
IGT: Impaired glucose tolerance
NDH: Non diabetic hyperglycaemia
NDPS: Norfolk diabetes prevention study
NHS: National Health Service
QALY: Quality adjusted life year
T2DM: Type 2 diabetes mellitus
TSC: Trial steering committee
