# Supplementary material for: Study Protocol: The Norfolk Diabetes Prevention Study [NDPS]: a 46 month multi - centre, randomised, controlled parallel group trial of a lifestyle intervention [with or without additional support from lay lifestyle mentors with Type 2 diabetes] to prevent transition to Type 2 diabetes in high risk groups with non - diabetic hyperglycaemia, or impaired fasting glucose
Source: BMC Public Health. 2017 Jan 6;17:31. doi: 10.1186/s12889-016-3929-5 (PMC5217324; doi:10.1186/s12889-016-3929-5)
Supplement: Additional file 1: — Description of intervention. (DOCX 252 kb) [file 12889_2016_3929_MOESM1_ESM.docx]

**The Norfolk Diabetes Prevention Study (NDPS) Lifestyle Intervention**

*Core development team:* The NDPS Lifestyle intervention was developed between June 2010 and May 2011 by a multi-disciplinary team, including health psychologists, physiotherapists, dietitians, a service user group and a diabetes consultant. The core team was:

Assoc. Prof Colin Greaves ^1^. Chartered Health Psychologist

Nikki Murray ^2^. Trainee Health psychologist.

Dr Alison Woodcock ^2^. Health and social psychologist (contracted consultant).

Prof Mike Sampson ^2^. Consultant Diabetologist, Chief Investigator for NDPS

^1^ Institute for Health Research, University of Exeter Medical School.

^2^  Norfolk and Norwich University Hospitals NHS Foundation Trust, Norwich, UK

A service user advisory group and a range of dietitians and physiotherapists were also consulted during the course of the development process (see Acknowledgements).

*Aims:* The intervention aims to reduce risk of type 2 diabetes in people with non-diabetic hyperglycaemia by encouraging weight loss, increasing physical activity, reducing intake of total and saturated fat and making other dietary changes (such as reducing portion sizes). . Behaviour change goals were set by participants but the NDPS intervention encouraged participants to shape these goals around 4 set targets; 1) If a BMI is over 30 kg/m^2^ then to work towards a 7% weight loss in the first 6 months and maintain until study end, 2) Reduce the amount of fat and specifically saturated fat from diet, 3) Work up to 150 minutes of moderate intensity physical activity on 5 days of the week or more which can be achieved through an increased step count and 4) work up to increasing muscle strength activity to 160-300 reps on 2-3 days of the week as these are known to induce clinically meaningful changes in diabetes risk for people with hyperglycaemia.

*Theoretical model:* We selected a theoretical basis for the intervention based on mapping recommendations for the content of diabetes prevention interventions^1^ (Table S1) and other evidence on behaviour change techniques or processes associated with effectiveness in changing diet and physical activity^2-4^, onto a range of possible theoretical models. The range of models considered was guided by the evidence based recommendations and included Self Determination Theory^5^, Social Cognitive Theory^6^, Control Theory^7^ and the Health Action Process Approach^8^. The Health Action Process Approach (HAPA) was considered to provide the “best fit” to the evidence-based recommendations. The HAPA proposes that initial motivation is mediated by self-efficacy, risk awareness and outcome expectancies. The following “volitional” phase involves action-planning, followed by action, and then further processes associated with maintenance of the new behaviour (primarily based on the facilitation of ‘learning from experience’). Self-efficacy is proposed to play a key role at each stage of the model. The HAPA was felt to provide a good fit to our identified needs in that a) it includes a strong self-regulatory component b) it has a strong focus on behaviour maintenance processes c) it addresses several key behavioural determinants and barriers identified in earlier assessments of the needs of people with pre-diabetes (e.g. perceptions of risk, self-efficacy)^9^.

| **Guideline recommendations** | **Intervention development & translation to model** |
| --- | --- |
| A clear plan of intervention should be developed, based on a systematic analysis of factors preceding, enabling and supporting behaviour change in the social /organizational context in which the intervention is to be delivered. **The plan should also identify the processes of change and the specific techniques and method of delivery designed to achieve these processes.** | Development followed the MRC framework for development of complex interventions to establish processes of change and specific techniques and delivery strategies to modify these processes. Social context is accounted for in exploring barriers to change. |
| **Use established well-defined behaviour change techniques.** There is some evidence suggesting that the following techniques may enhance effectiveness in physical activity and dietary interventions: Specific goal-setting, providing feedback on performance, self-monitoring, motivational interviewing, prompting self-talk and reviewing behavioural goals. |  |
| Providing instruction and the use of relapse prevention techniques (for dietary change), and prompting practice, individual tailoring, and time management (for physical activity), may also enhance effectiveness. | Established well-defined intervention techniques were identified based on existing interventions and a taxonomy of behaviour change techniques. Wherever, possible those with more established evidence that they enhance effectiveness (as opposite) were selected. |
| Dietary and /or physical activity interventions which **include prompting of self-monitoring alongside other self-regulatory techniques** (Specific goal setting; Prompting self-monitoring; Providing feedback on performance; Review of behavioural goals) may provide a good starting point for intervention design. | The intervention incorporates a number of self-regulatory techniques especially for behaviour maintenance. |
| **Include a strong focus on maintenance**. It is not clear how best to achieve behaviour maintenance but techniques designed to address maintenance include establishing self-monitoring of progress, providing feedback, reviewing of goals, engaging social support, use of relapse prevention /relapse management techniques and providing follow-up prompts. | The maintenance phase of the proposed intervention includes: self-regulation techniques (self-monitoring /feedback, goal review), reviewing of motivations and social support strategies, a strong emphasis on relapse prevention /relapse management (identification of barriers and problem-solving) and learning how to manage the process of change. We systematically considered emotional, cognitive, environmental and social barriers to maintenance. |
| **Maximize the frequency or number of contacts** within the resources available. | Working within the budgetary constraints for a pragmatic intervention, the use of group delivery will maximize the number of contacts (and experiential learning cycles) for each participant. |
| **Work with participants to engage social support** (i.e. to engage others who are important to them such as family, friends, and colleagues) in supporting the planned behaviour change | This is present at each stage of the model (motivation, action and maintenance). |
| **Aim to support changes in both diet and physical activity** | Behaviour change strategies to support change both diet and physical activity behaviours are present throughout the entire intervention |

**Table S1: Evidence based recommendations for content of interventions to prevent type 2 diabetes^1^.**

To address potential “gaps” in the HAPA model (areas where the existing HAPA does not explicitly address our evidence-based recommendations), we extended the model by adding a greater emphasis on social support, self-monitoring and relapse management and the use of relapse prevention (coping plans). Following a suggestion from our service-user group, we also added a novel element - explaining the process of behaviour change to participants. We also made adjustments to specify self-regulatory processes more clearly than in the original HAPA, by including self-monitoring, reviewing of progress (in terms of goals, motivation and social support) as well as relapse management processes (dealing with setbacks). The overall framework for the action and maintenance phases is therefore strongly based on self-regulation or ‘experiential learning’^10^.

The model was further extended by the specification of an over-arching aim: to empower participants (i.e. to increase their sense of ownership of behavioural goals and the process of achieving them and to give them the tools and skills needed to gain mastery over their eating /weight and physical activity /physical fitness). This was included to enhance patient engagement with the program and is consistent with the philosophy underlying interventions based on empowerment theory^11 12^ and with motivational interviewing, which our systematic review highlighted as a successful approach for initiating changes in dietary and physical activity behaviour^13 14^. Finally, to facilitate translation into a structured intervention programme, a focus on “processes of change”, rather than determinants was adopted.

The resulting theoretical model is shown in Fig.1. The model is deliberately expressed in lay language to facilitate its use as an intervention tool (to aid understanding of the process of behaviour change). The key intervention processes are i) increasing motivation (perceived importance of healthy lifestyle, self-efficacy for achieving healthy lifestyle, perceived risk and outcome expectations); ii) making a specific action plan (including plans for social support and for overcoming barriers (coping plans)) and iii) supporting maintenance through repeated ‘self-regulatory cycles’ of planning, self-monitoring and other feedback (e.g. on blood sugar), problem-solving to manage setbacks and revision of action plans. There is also a strong emphasis on empowering participants to develop autonomous motivation and to “make changes you can live with” to ensure that plans for lifestyle change are sustainable.


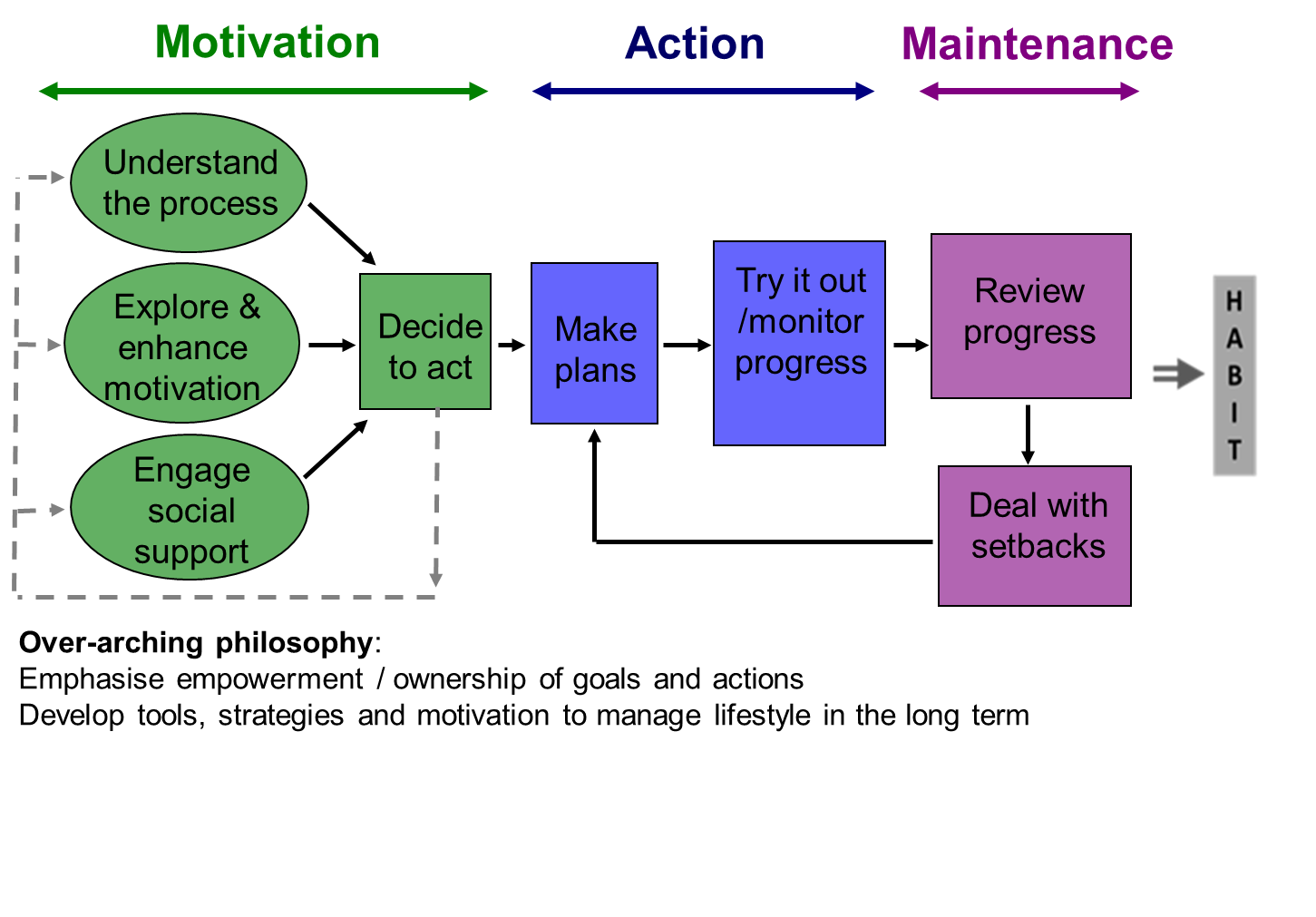


**Fig.1: The Process Model of Lifestyle Behaviour Change^15-17^**

It is worth noting that the HAPA motivational determinants “risk awareness”, “outcome expectancies” and “perceived self-efficacy”, although not shown in the figure, are included in the fully specified model, but have been subsumed within the broader heading "explore and enhance motivation". The detailed content of the action planning phase (specifying behaviour changes; specifying coping strategies (relapse prevention) and addressing social influences) are also not shown in the figure. The specification of key topics for review at the maintenance stage (social influences and motivation) are also not shown in the figure.

***Content:***

The behaviour change techniques used to promote changes in each of the targeted processes are illustrated in Fig S2. These were specified by a) reviewing intervention techniques and strategies used in successful behaviour change interventions for dietary and physical activity behaviours, identified through references in relevant high quality systematic reviews and clinical guidelines^14 18-20^ and through consultation with experts in the field and b) referring to a taxonomy of behaviour change techniques^21^ c) ideas and adaptations suggested by the intervention development working group. These included motivational interviewing, self-assessment of risk factors (diet and physical activity levels), identifying and engaging sources of social support /addressing social influences, SMART-ER goal setting (Specific, Measurable, Achievable, Relevant, Time-related, Enjoyable, Repeatable), making coping plans and social support plans, self-monitoring of outcomes (using self-weighing and the participants’ choice of pedometers or physical activity diaries), frequent reviews of progress, problem-solving and goal review. We had a strong emphasis on empowering participants to develop and practice skills for lifestyle behaviour change. This included discussing how the process of behaviour change works and why they were being asked to engage in the different activities (such as action planning and self-monitoring). Thus, we made the model we were using (Fig.1) explicit from the outset.


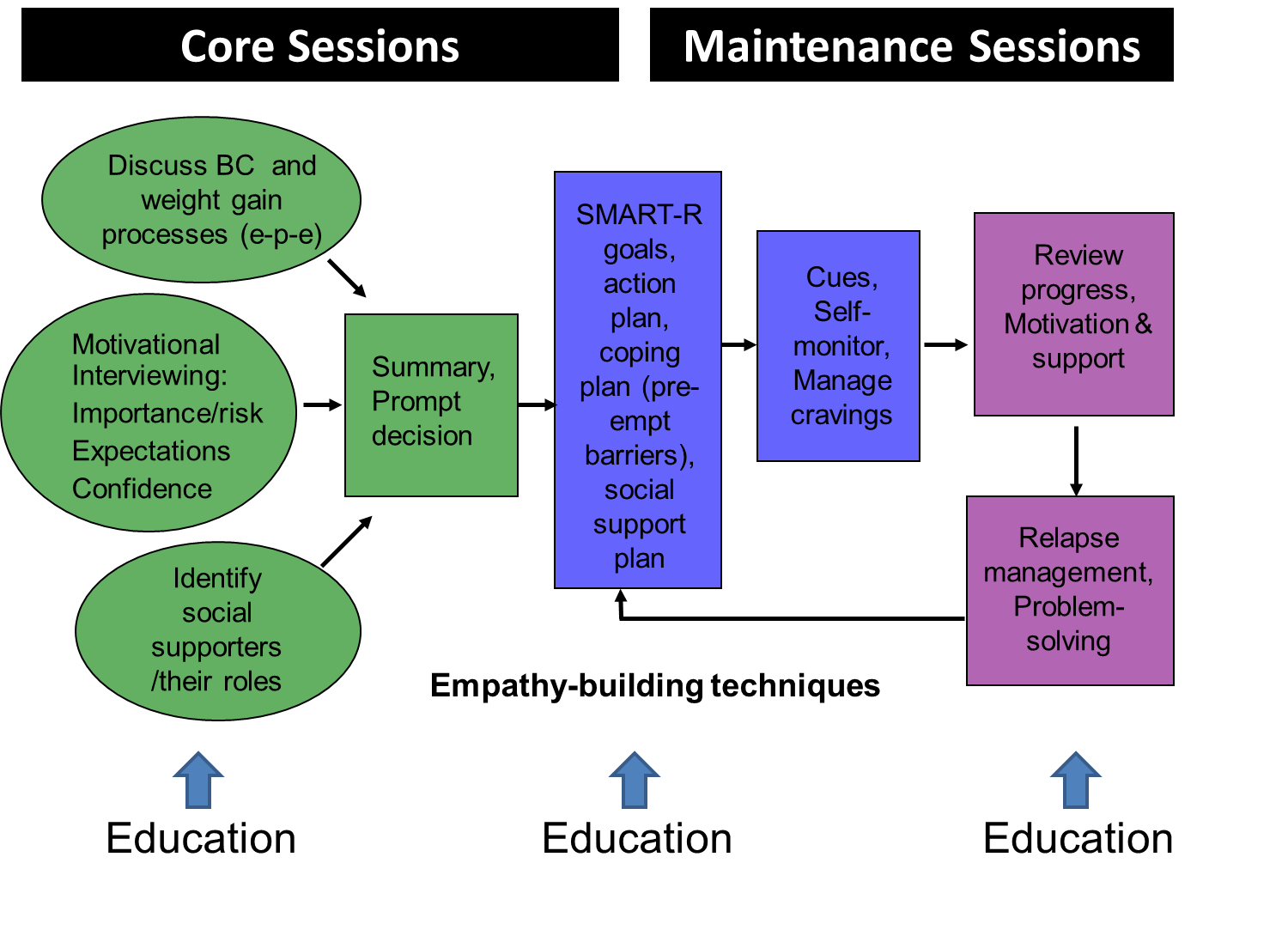


**Fig.S2: Behaviour change techniques used in Waste the Waist**

To promote sustainability of weight loss we advised participants to make a series of small, achievable changes, rather than dramatic, unsustainable changes.^20^ We encouraged participants to prioritise ideas for change that would not detract from their enjoyment of food (for dietary changes) or that would be enjoyable or easy to build into a routine (for physical activity)^22^. Key messages that were repeated throughout the programme were “Make changes you can live with”, “Small changes make a big difference” and “Aim for a lifestyle that is both healthy and enjoyable”. We also included one technique based on cognitive behavioural therapy (using a mental ‘STOP’ sign and some pre-conceived strategies to address ‘in the moment’ food cravings, which were identified by our service users as a key barrier to success in changing eating behaviour).

Group delivery was chosen to maximize the number of contacts and contact time for each participant within the budgetary constraints of a pragmatic intervention. However, the choice was based on more than simple cost considerations. It was considered that working in groups a) allows for sharing of ideas and knowledge across a wide pool of expertise (from the participants) b) allows the development of a group identity (having a common mission /being in the same boat) which, in theory should increase motivation to attend (assuming the group identity is viewed positively) as well as to make changes c) maximises the number of experiential learning cycles that can be completed by each person d) provides a source of “accountability” that could add to motivation for weight loss^23^.

Throughout the intervention we provided and discussed information about

1. Healthy eating - we used the Public Health England “Eat Well Guide”^24^ as a guide and intervention tool to prompt ideas for reducing calorie intake by replacing fat (especially saturated fat) with fruit, vegetables and high fibre carbohydrates, reducing intake of high fat /high sugar snacks and reducing overall calorie intake.
2. Physical activity – including how to gauge intensity, especially in relation to walking pace and discussing ideas and opportunities for increasing physical activity. A set of home-based resistance exercises (as per government guidance to engage in muscle-strengthening exercise involving all muscle groups on at least two days of the week^25^) was introduced and discussed. Demonstration /reinforcement of these principles using structured exercise classes was provided during the maintenance sessions.

Practical intervention strategies and intervention materials were developed by the intervention development working group to implement the selected behaviour change techniques and organised into a framework for program delivery. The process model was particularly relevant here in guiding the sequence of delivery of the selected strategies. Detailed session plans specifying a comprehensive practical intervention plan and a set of slides to facilitate delivery were then created.

*Structure:* The NDPS Lifestyle Intervention begins with six two-hour group based education /behaviour change intervention sessions spread over 12 weeks, After a four week gap, this is followed by up to 15 2.5-hour group based maintenance sessions (including behaviour maintenance techniques and one hour of structured exercise) delivered every 8 weeks (see Fig.2 in the main paper for session timings). The initial group size is 10-12 participants, with options to merge groups over time (at the maintenance stage) if attendance diminishes. The total contact time (assuming all maintenance sessions are attended) is therefore 49.5 hours (including 15 hours of structured physical activity) spread over 30 months.

*Delivery and facilitator training:* The initial six session “core” intervention is delivered by a single facilitator. Where possible, the same facilitator delivers all sessions for a given group of participants (although this is not always possible). Tables are arranged in a “U-shaped” (horseshoe) configuration with the facilitator and slide projection area at the open end. The sessions are semi-structured with a printed session plan and a set of Powerpoint slides for each session. Presentation of the session is driven from the slides (using icons to remind the facilitator to ‘pause’ for planned interactive activities), with a mixture of didactic presentation (providing information) and interactive activities (slides are paused and participants engage in a discussion or other interactive activity, such as problem-solving, or action-planning). Content is tailored to build on the existing knowledge and skills of participants and the facilitator spends individual time with individuals who need help during action-planning activities and tries where possible (within the limits of group-based delivery) to elicit and respond to individual motivations, barriers to change or other concerns. Flipcharts were used to keep track of ideas from the group in a number of interactive activities and additional printed information materials (handouts) were available for a number of sessions covering a range of topics (e.g. healthy eating, why weight loss plateaus and what to do about it, a pictorial diagram of a set of muscle-strengthening exercises that can be performed at home /without any equipment). The maintenance sessions are delivered by a NDPS facilitator (as above) and the training and background of these staff are described in the main paper.

A five day training course was developed (3 days on core education sessions and two days on maintenance sessions) and delivered by the co-authors (primarily NM, CG, SA). The course content (see Supplementary File 2) focuses on developing behaviour change skills (Box 1), updating knowledge (e.g. on healthy eating), using the intervention materials, delivering the session content using the slides and session plans and data collection issues (including providing data for the research study). The training for DPFs and physiotherapists covers group dynamics, including factors that define a ‘successful’ group (appropriate leadership, common goals, facilitative group climate, mutual respect and group attractiveness (including having fun, positive attributions about the social experience) and how group dynamics develop (Forming, Storming, Norming and Performing).

The style of delivery is important (both for personal engagement /motivation and for promoting positive group dynamics) and we trained the facilitators to use person-centred counselling techniques based on motivational interviewing (open questioning, affirmation, reflective listening, summaries, use of the Ask-Tell-Discuss technique for information exchange)^26 27^] to promote autonomous motivation and to deliver all of the intervention content. A selection of 3-4 audio recordings per facilitator of session delivery is reviewed by the trainers to check fidelity of intervention delivery and facilitators also self-complete a fidelity checklist at the end of each session. The trainers use this data to a) develop training updates and b) give the facilitators individual formative feedback. Supervision meetings are held approximately every two months, where barriers and solutions to delivery are discussed. The intervention is delivered in local community venues (e.g. community halls, meeting rooms in GP practices after hours, University premises).

| 1. Engaging the patient - put them in charge, develop their interest and motivation - (MI, Reflective listening, Affirmation, Rolling with Resistance) 2. Exchanging information: Ask – Tell - Discuss 3. Exploring the ‘importance’ of making changes    1. make the link between lifestyle behaviours and heart health    2. help them to 'weigh up' the advantages and disadvantages (pros and cons) of making changes 4. Problem-solving (identifying and finding ways to overcome individual barriers) 5. Building confidence (gradual accumulation of knowledge and mastery of lifestyle behaviours) – use the Confidence Ruler and If-Then (coping) plans 6. Making the group work well |
| --- |

**Box 1: Behaviour change skills for delivery of the NDPS Lifestyle Intervention**

**Acknowledgments**

We are grateful to colleagues who contributed to the development of these and associated materials

Mrs Sara Auckland Norfolk and Norwich university Hospital

Mrs Sue Baic University of Bristol

Emma Cooper Norfolk and Norwich university Hospital ?ossibly Gea? Fiona Gillison (UNillison (UNiversity of Bath)

Dr Afroditi Stathi University of Bath

Dr Fiona Gillison University of Bath

**References**

1. Paulweber B, Valensi P, Lindström J, et al. A European evidence-based guideline for the prevention of type 2 diabetes. *Hormone and Metabolic Research* 2010;42:S3-S36. doi: 10.1055/s-0029-1240928

2. Greaves CJ, Sheppard KE, Abraham C, et al. Systematic review of reviews of intervention components associated with increased effectiveness in dietary and physical activity interventions. *Bmc Public Health* 2011;11(119):1-12. doi: 10.1186/1471-2458-11-119

3. Dombrowski SU, Sniehotta FF, Avenell A, et al. Identifying active ingredients in complex behavioural interventions for obese adults with obesity-related co-morbidities or additional risk factors for co-morbidities: a systematic review. *Health Psychology Review* 2012;6(1):7-32.

4. Artinian NT, Fletcher GF, Mozaffarian D, et al. Interventions to promote physical activity and dietary lifestyle changes for cardiovascular risk factor reduction in adults: A scientific statement from the American Heart Association. *Circulation* 2010;122(4):406-41.

5. Deci EL, Ryan RM. Intrinsic motivation and self-determination in human behavior. New York: Plenum Publishing Co 1985.

6. Bandura A. Social foundations of thought and action: A social cognitive theory. Englewood Cliffs, NJ: Prentice-Hall 1986.

7. Carver CS, Scheier MF. Attention and self-regulation: A Control-Theory Approach to Human Behavior. New York: Springer-Verlag 1981.

8. Schwarzer R. Self-efficacy in the adoption and maintenance of health behaviors: Theoretical approaches and a new model. In: Schwarzer R, ed. Self-efficacy: Thought control of action. Washington DC: Hemisphere Publishing Corp 1992:217–43.

9. Evans PH, Greaves C, Winder R, et al. Development of an educational "toolkit" for health professionals and their patients with prediabetes: The WAKEUP study (Ways of Addressing Knowledge Education and Understanding in Pre-diabetes). *Diabetic Medicine* 2007;24(7):770-77. doi: 10.1111/j.1464-5491.2007.02130.x

10. Kolb DA. Experiential learning experience as the source of learning and development. Englewood Cliffs, NJ: Prentice-Hall 1984.

11. Funnell MM, Anderson RM, Arnold MS, et al. Empowerment: an idea whose time has come in diabetes education. *The Diabetes educator* 1991;17(1):37-41.

12. Skinner TC, Cradock S, Arundel F, et al. Four theories and a philosophy: Self-management education for individuals newly diagnosed with type 2 diabetes. *Diabetes Spectrum* 2003;16(2):75-80.

13. Burke B. Motivational interviewing: A meta-analysis of controlled clinical trials. *Dissertation Abstracts International: Section B: The Sciences and Engineering* 2004;64(9-B):4605.

14. Rubak S, Sandboek A, Lauritzen T, et al. Motivational interviewing: a systematic review and meta-analysis. *British Journal of General Practice* 2005;55:305-12.

15. Greaves CJ, Reddy P, Sheppard K. Supporting behaviour change for diabetes prevention. In: Schwarz P, Reddy P, Greaves CJ, et al., eds. Diabetes Prevention in Practice. Dresden: TUMAINI Institute for Prevention Management 2010:19-29.

16. Greaves CJ. Supporting behaviour change in general practice. In: Barnard K, Lloyd CE, eds. Practical Psychology in Diabetes Care. London: Springer-Verlag 2012.

17. Lindström J, Neumann A, Sheppard K, et al. Take Action To Prevent Diabetes: A toolkit for the prevention of type 2 diabetes in Europe. *Hormone and Metabolic Research* 2010;42:S37-S55. doi: 10.1055/s-0029-1240975

18. Avenell A, Broom J, Brown TJ, et al. Systematic review of the long-term effects and economic consequences of treatments for obesity and implications for health improvement. *Health Technology Assessment* 2004;8(21)

19. Shaw K, Rourke P, Del M, et al. Psychological interventions for overweight or obesity. *Cochrane database of systematic reviews (Online)* 2005(2):CD003818.

20. Excellence. NIfHaC. Obesity guidance on the prevention, identification, assessment and management of overweight and obesity in adults and children. London, 2006.

21. Abraham C, Michie S. A taxonomy of behavior change techniques used in interventions. *Health psychology : official journal of the Division of Health Psychology, American Psychological Association* 2008;27(3):379-87. doi: 10.1037/0278-6133.27.3.379

22. Davis M, Fox K, Hillsdon M, et al. Getting out and about in older adults: the nature of daily trips and their association with objectively assessed physical activity. *International Journal of Behavioral Nutrition and Physical Activity* 2011;8(1):116.

23. Metzgar CJ, Preston AG, Miller DL, et al. Facilitators and barriers to weight loss and weight loss maintenance: a qualitative exploration. *Journal of Human Nutrition and Dietetics* 2014;28(6):593-603

24. Eat well guide. Department of Health Public Health England. Published 17^th^ March 2016 Accesssed 21.3.16 http://www.nhs.uk/Livewell/Goodfood/Pages/the-eatwell-guide.aspx

25. Health Do. Start Active, Stay Active: A report on physical activity for health from the four home countries' Chief Medical Officers. London, 2011.

26. Miller WR, Rollnick S. Motivational interviewing: Preparing people for change (2nd ed). New York: Guildford Press 2002.

27. Rollnick S, Mason P, Butler C. Health Behaviour Change: A guide for practitioners. Edinburgh: Churchill Livingstone 1999.
