# Supplementary material for: Study Protocol: The Norfolk Diabetes Prevention Study [NDPS]: a 46 month multi - centre, randomised, controlled parallel group trial of a lifestyle intervention [with or without additional support from lay lifestyle mentors with Type 2 diabetes] to prevent transition to Type 2 diabetes in high risk groups with non - diabetic hyperglycaemia, or impaired fasting glucose
Source: BMC Public Health. 2017 Jan 6;17:31. doi: 10.1186/s12889-016-3929-5 (PMC5217324; doi:10.1186/s12889-016-3929-5)
Supplement: Additional file 2: — DPF Training manual. (DOCX 309 kb) [file 12889_2016_3929_MOESM2_ESM.docx]

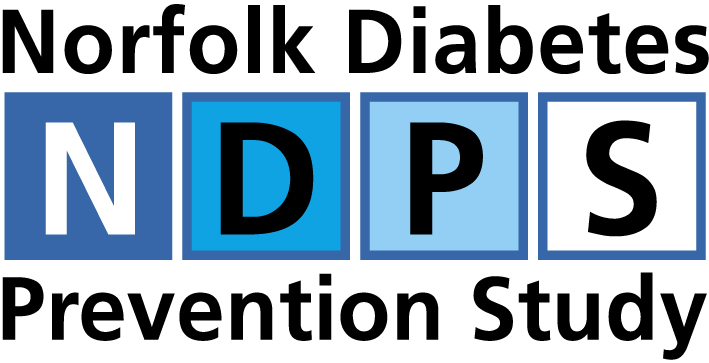


**Intervention Staff**

**Training Document for ‘Trainers’**

**
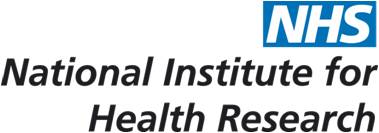
**

**
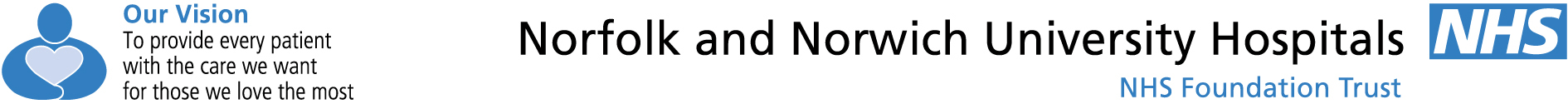
This manual has been prepared by:**

Nikki Murray BSc (Hons), MSc

Colin Greaves, PhD, C Psychol

Sara Auckland (BSc (Hons), PhD

On behalf of the Norfolk Diabetes Prevention Study

**Approved by:**

**Name: Role:**

**Signature: Date:**

**Authorised by:**

**Name: Role:**

**Signature: Date:**

# Contents Page

[Contents Page 3](#_Toc330278182)

[How to use this manual 5](#_Toc330278183)

[Chapter 1: Training Overview for all Intervention Staff 6](#_Toc330278184)

[Chapter 2: Introduction to Research 7](#_Toc330278185)

[Chapter 3: Introduction to the office 8](#_Toc330278186)

[Chapter 4: NNUH Mandatory training 9](#_Toc330278187)

[Chapter 5: Introduction to the NDPS Documentation 10](#_Toc330278188)

[Chapter 6: Worldwide Diabetes Prevention Programme Trials 11](#_Toc330278189)

[Chapter 7: Diabetes Information 12](#_Toc330278190)

[Chapter 8: Control Session Training 13](#_Toc330278191)

[Chapter 9: Education Session Training for DPFs 14](#_Toc330278192)

[Education Session Plans 16](#_Toc330278193)

[Chapter 10: Maintenance Session Training 17](#_Toc330278194)

[Chapter 11: Action Plans and Progress Review Sheets 19](#_Toc330278195)

[Action Plans 19](#_Toc330278196)

[Progress Review Sheets 20](#_Toc330278197)

[Chapter 12: Process Analysis and Intervention fidelity 21](#_Toc330278198)

[Chapter 13: Randomisation and Consent Training 22](#_Toc330278199)

[Tanita BC-420MA Body Composition Analyser 25](#_Toc330278200)

[Chapter 14: Nutrition Training 27](#_Toc330278201)

[Chapter 15: Behaviour Change Training 28](#_Toc330278202)

[Key Skill 1: 32](#_Toc330278203)

[Key Skill 2: ELICIT 🡪 PROVIDE 🡪 ELICIT 33](#_Toc330278204)

[Key Skill 3 - Importance 34](#_Toc330278205)

[Key Skill 4 - Problem Solving-Decisional Balance 35](#_Toc330278206)

[Key Skill 5: Confidence 36](#_Toc330278207)

[Key Skill 6 – Group Dynamics 37](#_Toc330278208)

[What makes a group work? 37](#_Toc330278209)

[Chapter 16: Shadowing, Practice and Assessment 39](#_Toc330278210)

[Chapter 17: Refresher Training 42](#_Toc330278211)

[Agenda for DPF Education Training Day 1 43](#_Toc330278212)

[Agenda for DPF Education Training Day 2 44](#_Toc330278213)

[Agenda for DPF Education Training Day 3 45](#_Toc330278214)

[Agenda for Physio Education Session Training Day 46](#_Toc330278215)

[Agenda for Day 1 47](#_Toc330278216)

[Agenda for Day 2 48](#_Toc330278217)

[Agenda for Physiotherapist Maintenance Session Training 48](#_Toc330278218)

[9.4 Motivational Interviewing Workshop 50](#_Toc330278219)

[9.7 Ground Rules 50](#_Toc330278220)

[9.8 Participant behaviour 51](#_Toc330278221)

[9.9 What do we need to be able to do to make a successful group experience? 51](#_Toc330278222)

[9.10 Open questions 52](#_Toc330278223)

[9.11 Explanatory Questions 52](#_Toc330278224)

[9.12 Word Reflections 52](#_Toc330278225)

[9.13 Non-Verbal Skills 53](#_Toc330278226)

[9.14 Goals 53](#_Toc330278227)

[9.15 Action Planning 53](#_Toc330278228)

[9.17 Support Self-Efficacy 54](#_Toc330278229)

[9.19 Cost Benefit Analysis 54](#_Toc330278230)

[9.20 Look out for the signs 55](#_Toc330278231)

[Chapter12: WORKSHEETS 56](#_Toc330278232)

[Worksheet 1: Reflections worksheet 56](#_Toc330278233)
