# Supplementary material for: Study Protocol: The Norfolk Diabetes Prevention Study [NDPS]: a 46 month multi - centre, randomised, controlled parallel group trial of a lifestyle intervention [with or without additional support from lay lifestyle mentors with Type 2 diabetes] to prevent transition to Type 2 diabetes in high risk groups with non - diabetic hyperglycaemia, or impaired fasting glucose
Source: BMC Public Health. 2017 Jan 6;17:31. doi: 10.1186/s12889-016-3929-5 (PMC5217324; doi:10.1186/s12889-016-3929-5)
Supplement: Additional file 3: — Protocol version tracker. (DOCX 24 kb) [file 12889_2016_3929_MOESM3_ESM.docx]

**Appendix 1.0**

**NDPS Protocol change control, revision and review Sheet**

| Version No | Change Date | Reason for Change |
| --- | --- | --- |
| Version 2.0 | 03/05/2011 | All the modified amendments have been highlighted. The ‘strike through’ tool has been used to indicate the removal of text. The amendments DO NOT significantly alter the research design or methodology and will not affect the scientific value of the study.  Correction of grammatical errors  Change of staff names  An additional collaborator added |
| Version 3.0 | 24/06/2011 | (P33) text regarding randomisation has been re-worded for clarity |
| Version 4.0 | 25/07/2011 | been re-worded for clarity and punctuation/spelling mistakes have been amended |
| Version 5.0 | 18/07/2012 | These proposed amendments are:  a) We are now recruiting from the GP practices in Great Yarmouth, and screening these participants at the James Paget Hospital in Great Yarmouth. We would now also like to recruit from practices in the nearby towns, such as Beccles, Bungay and Lowestoft that use the James Paget Hospital and which have large populations. In addition, we will be screening at Thetford, (Norfolk), and would like to offer patients from nearby towns such as Brandon the opportunity to take part. The practices’ populations in these nearby towns are at least partly in Suffolk, and we would like to amend the PIS to remove the section that says participants must ‘live in the County of Norfolk’. We think this would be equitable so that all potential participants who can access the programme at a Norfolk site can do so. We attach a revised PIS with these and other minor changes tracked in b) The main recruitment strategy in this programme is to write to potential participants with the approved PIS, having undertaken a GP database search for suitable populations. The response to these large mailshots has been variable, with 5 – 20% of invited participants agreeing to screening by making direct contact with the study team. To enhance recruitment, we would now like to send a short follow up letter to the large population of non-responders who are still at high risk of T2DM, reminding them of the opportunity to take part in the programme should they wish. The follow up letter would be sent no less than four weeks after the initial invitation letter. We attach a copy of the proposed reminder letter, and have kept this as neutral as possible, and we would not send the detailed PIS again with this letter.  We would like to do this retrospectively to the practices we have already visited, and prospectively in the practices we have prepared. We think this is equitable and offers the programme to those who didn’t or couldn’t respond at first contact, but who still may wish to take part. We would do this after discussion with individual practices, and we would target those where the response was lowest |
| Version 6.0 | 03/12/2012 | Addition of the Episwitch Study; The NDPS screens between 50 – 90 subjects per week (mean age 58 years), the participants undergo a fasting glucose and HbA1c analysis and will generate a sample size of 10,000. We propose to take a further 6mls EDTA blood sample from 300 consecutive participants (approximately over a 4 week period) during normal trial recruitment in addition to routine study sample collection. The PIS and consent form have been amended to highlight this extra sample taken at screening and the reason for the sample has been explained in the lay person. This n=300 sample (based on current data from about 3500 patients) would generate group sizes of approximately  1) 45 subjects with normal fasting glucose, but ‘pre diabetes’ glycated haemoglobin HbA1c (42 – 48 mmol/mol)  2) 15 subjects with impaired fasting glucose (6.1 – 6.9 mmol/l)  3) 15 subjects with T2DM ( based on HbA1c > 48 mmol/mol or fasting plasma glucose > 7 mmol/l)  4) 225 subjects with normal fasting glucose (< 6.1 mmol/l) and HbA1c (< 42 mmol/mol)  We would suggest analysis on groups 1 – 3 above and on 60 randomly selected age matched controls from the normal glucose regulation group (group 4). |
| Version 7.0 | 16/01/2013 | This amendment is a revised version of Substantial Amendment 27, submitted on the 3rd of December 2012. In accordance with the Committee's guidance the participants who provide the extra sample will do voluntary without affecting eligibility otherwise for the study. The documentation has been amended to reflect this. The text in the protocol and PIS has been amended to highlight this sampling is optional and a separate consent form has been developed. |
| Version 8.0 | 16/06/2013 | HbA1c Observational study  In summary, the main issue is that in 2012 the diagnostic criteria for diabetes were changed, and these changes are now adopted in the UK and internationally. These new criteria are based on measurement of the HBA1c (a blood test that gives an estimate of the average glucose level over the previous 8 – 12 weeks). These new HbA1c criteria (which do not exclude a glucose based diagnosis), indicate a diagnosis of diabetes when the HbA1c is >48 mmol/l , and also now create an entirely new group of people with ‘pre diabetes’ based on having an HbA1c level of 42 – 47 mmol/mol (inclusive), who are at increased risk of progressing to diabetes. In the main NDPS study, we have found so far about 350 people of the 5000 screened have this new category of ‘pre diabetes’ but also have had a normal fasting plasma glucose . These people have been told that they have a normal fasting glucose in line with REC approved materials, but this new diagnostic criteria (2012) for diabetes now retrospectively puts these people , unexpectedly, into a prediabetes category. We feel that ethically we need to let these participants know about this change, and to retrospectively (and prospectively) offer entry into the main programme. The unusual issue with this group (raised HbA1c but normal fasting glucose) is that it is entirely unknown what their chance of progression to T2DM is, or how diet and lifestyle interventions could influence this. This proposed amendment would have very significant research value in answering these questions, and also offer participants in this new category the chance to enter the programme. If participants decline to be screened or randomised we will ask them to make contact with their GP for repeat screening and we will communicate this with the GP. There are no data that would allow power calculations for this group, and we would like to randomise these subjects(we estimate perhaps 200 would consent) into an observational cohort within NDPS randomised to a control or intervention group, and would like to approach those already screened , and those found to have this abnormality in the remaining screening period. We have discussed with this the NIHR Programme Management team and have secured agreement to proceed with this subject to Ethical review; I attach a summary of this application to NIHR which gives more background. Finally, a small number of people we have screened have had a normal fasting glucose and yet have an elevated hbA1c in the diabetes range (> 48 mmol/mol) and we have drafted letters to these participants offering to repeat these measurements or ask GP practices to do this. |
| Version 9.0 | 15/07/2013 | With reference to NOSA 30 above we would like to test for haemoglobin and Ferritin levels in participants of the Prediabetes HbA1c study. The HbA1c fraction is abnormally elevated in chronic hyperglycaemic diabetic patients and correlates positively with glycaemic control. Previous studies suggest that iron deficiency anaemia affects the levels of HbA1c. Serum Ferritin is a marker of iron stores in the body. It has been suggested that iron deficiency must be identified and corrected before any diagnostic decision is made based on HbA1c. Therefore we would like to test the levels of Ferritin alongside the HbA1c tests in these participants |
| Version 10.0 | 10.0, 10.1, 10.2 | In house working updates in preparation for version 11 submission (not submitted to REC) |
| Version 11.0 | 03/04/2014 | Diagnostic cut point of plasma glycosylated haemoglobin (HbA1c) ≥48 mmol/mol for the diagnosis of diabetes (and by extension categorisation of prediabetes) was supported by the American Diabetes Association (ADA), the International Expert Committee (IEC) and by the WHO, between 2010 – 2011. This strategy is now being widely adopted in UK primary care, at least in many CCG areas in England, and reinforced by NICE guidance in mid2012. In NDPS we decided to measure HbA1c at all-time points at screening and intervention, at the same time as a fasting plasma glucose, as we were aware of these possible diagnostic changes at the time of award (2010). The move of practices to diagnosis based on HbA1c rather than glucose criteria (although this is not possible to quantify) has reduced the numbers of subjects with impaired fasting glucose ( IFG) on practice registers (by diagnostic category or glucose data), as many subjects with IFG would fall into the new diabetes categories based on HbA1c. There is still very substantial value in fasting glucose data (combined with pre diabetes HbA1c values), as a marker of increased risk of progression to T2DM. In 2013 we were granted approval for an additional add on study which would go some way in addressing issues raised in the above national guidance on prediabetes. This study population (n = 200) have a normal fasting plasma glucose (NFG; < 6.1 mmol/l), but who fall into the new diagnostic category of prediabetes based on an elevated HbA1c value of ≥42 47 mmol/mol inclusive. The accrual for this trial is almost complete. This cohort of participants have a higher prevalence (10.1%) of impaired fasting glucose or IFG (6.1 – 6.9 mol/l) than participants recruited via our current methods (5.9%). We would now like to incorporate the new diagnostic category of prediabetes based on an elevated HbA1c value into our GP database search criteria as this population seem a rich source of IFG (6.1 – 6.9 mmol/l) subjects. The inclusion/exclusion criteria would remain the same as the term ‘prediabetes’ is used throughout our documents, as opposed to the term ‘IFG’, the term prediabetes with cover all prediabetes regardless of which test was used to determine this. We ask for approval for the search criteria for the GP surgeries to run to be as follows. Currently the search criteria reads as follow:  . Parent, sibling or child with T2DM  ∙ Personal history of coronary disease  ∙ Previous history of gestational diabetes  ∙ Known impaired fasting glucose (IFG) or impaired glucose tolerance (IGT)  ∙ Fasting glucose range of 6.1 – 6.9mmol/l)  ∙ BMI ≥30kg/m2  We ask for approval for the search criteria for the GP surgeries to run to be as follows  . Parent, sibling or child with T2DM  ∙ Personal history of coronary disease  ∙ Previous history of gestational diabetes  Notice of Amendment IRAS  ∙ BMI ≥30kg/m2  We ask for approval for the search criteria for the GP surgeries to run to be as follows  . Parent, sibling or child with T2DM  ∙ Personal history of coronary disease  ∙ Previous history of gestational diabetes  ∙ Known impaired fasting glucose (IFG) or impaired glucose tolerance (IGT)  ∙ Fasting glucose range of 6.1 – 6.9mmol/l)  ∙ HbA1c value of ≥42 47  mmol/mol inclusive  ∙ BMI ≥30kg/m2  We ask for approval for the search criteria for the GP surgeries to run to be as follows  . Parent, sibling or child with T2DM  ∙ Personal history of coronary disease  ∙ Previous history of gestational diabetes  ∙ BMI ≥30kg/m2  We ask for approval for the search criteria for the GP surgeries to run to be as follows  . Parent, sibling or child with T2DM  ∙ Personal history of coronary disease  ∙ Previous history of gestational diabetes  ∙ Known impaired fasting glucose (IFG) or impaired glucose tolerance (IGT)  ∙ Fasting glucose range of 6.1 – 6.9mmol/l)  ∙ HbA1c value of ≥42 47  mmol/mol inclusive  ∙ BMI ≥30kg/m2  Amendment 2  To maintain power in the programme we would like to widen recruitment into the diabetes prevention studies from new populations with prediabetes at equivalent high risk of progressing to diabetes. This would be in line with recent NICE guidance, and changes in NHS policy in the detection of prediabetes. In addition, present recruitment policies would only reach a final sample size of 650 in the diabetes prevention projects, less than the required 950 sample, which would have significant impact (clinical, research, and ethical) on the value of the programme unless we reach final sample size. This new large prediabetes populations are those who are at equivalent high risk of transition to T2DM as our recruited participants with a FPG of >6.1 – 6.9 mmol/L. Based on existing NDPS infrastructure and policies, we would like to ask approval to recruit subjects with a high risk combination of prediabetes HbA1c (42 – 47 mmol/mol) and IFG (5.6 – 6.0 mmol/l) fasting plasma glucose. This approach will be applied in 75 existing practices in Norfolk and Suffolk. This lower glucose range (5.6 – 6.0 mmol/l) category is also recognised as impaired fasting glucose.  One further amendment we would like to ask approval for is to be able to offer the use of Docmail alongside our standard procedure of mail out. As we have almost completed the Norfolk mail outs this will predominantly be used/promoted in the Suffolk area. Docmail is an online mail management system for letter printing and mailing needs. The letter and PIS/approved documents are uploaded by the study team in Microsoft Word format (.doc), as an Adobe Acrobat file (.pdf) or in rich text format (.rtf) and the details of the GP patients plus their standard GP letter template will be uploaded via the practice staff. Docmail will allow the practice to enter mailing requirements via a website to our secure servers. Docmail is delivered to us direct by CFH Total Document Management Limited (CFH), one of the UK’s leading document outsource processors. The latest printing and enclosing technology combined with envelope sorting equipment is used. These tools will provide considerable savings to the project whilst allowing the letters to be personalised to the patient and also enable the GP logo along with the study logo to be incorporated in the letter head. The Docmail website uses the highest strength 128 bit encryption and many GP surgeries are already using this secure system to send letters to their patients. The CFH production site assures customers that they have the highest levels of both physical and IT security which enables us to process mailing with complete assurance.  Finally we would like to ask for permission to extend the follow up period from 40 months to 46 months. This approach suggests re-consenting all in trial subjects to extend follow up and exit from Projects 2 and 3 from 40 months to 46 months, and for new randomisations to be consented on the basis of 46 months follow up. This is necessary to maintain power, and subjects already in the programme who do not wish to do this will be followed as already consented to 40 months. This single additional visit would, based on a randomisation rate of 13.5 subjects per week for 52 weeks, generate a final sample size of 1031. Assuming a 14.5% withdrawal rate with mean withdrawal at 12 months in those who withdraw, we would have 995 individuals with exit data and a mean follow up of 38.4 months. This is based on the assumption that the programme extends from March 2016 to March 2018 to allow 52 weeks further screening and randomisation and minimum follow up of 36 months for all new accruals until March 2016. This sample would generate in programme transition to T2DM rates (over the intervention period) modelled at 23.4 % in the controls, 12.2% in the intervention group and 6.3% in the intervention + diabetes prevention mentors (DPM) group. The 995 individuals would be randomised as 199 in the control group, 398 in the intervention and 398 in the intervention + DPM group. The impact of this would give 91% power to detect this effect size in control vs intervention (Project 2) ; >99% power for control vs >intervention + the diabetes prevention mentors DPM (Project 3); and 80 % power to detect this effect size between the two intervention groups (intervention with or without the DPM; Projects 2 and 3).  Added text and information page 60 Quality Assurance/Audits/Quality Control of Documents. The programme will receive formal quality assurance support from the Norwich CTU including the documentation of all quality procedures in a Quality Management Plan and assistance to set up the TSC and DMC with roles and responsibilities of these committees documented in line with established guidelines. |
| Version 12.0 | 10/06/2014 | There are three PhD projects attached to this programme. The full details of all PhD's have been previously submitted and fully approved. Including all documentation associated with the projects. However it has been noted that the details of the projects have not been fully explained in the Protocol. We wish to re-submit the protocol with fuller details of the two projects explained in more detail.  Submission of participant’s letters. S20 - this letter will advise the participant that they have a confirmed prediabetes result confirmed with the HbA1c test only and a normal fasting glucose result. However as the trial is fully recruited we will not be able to offer the intervention trial. S26 is the participants GP letter. S88 is a generic second appointment letter. |
| Version 13.0 | 02/09/2014 (submitted 30/01/2015) | The NIHR raised uncertainty around the sample size calculation of project 4 (screen detected T2DM project). Due to the inclusion of pilot study patients and the differences in the timeframe of delivery of the intervention and length of follow up between the pilot and main trials (newly recruited patients receive all the education sessions of the intervention consecutively while pilot patients would be given maintenance sessions only, having already received the first education during the pilot study undertaken some time ago). Whilst 13 pilot study participants have consented for the NDPS, the inclusion of these participants is not required to power the study providing sufficient numbers of patients (100 for each arm) are recruited to maintain statistical power. Therefore we asked permission to increase the number recruited in to project 4 from 240 to 300.  The enhanced the power of the study by offering extended follow up in the Project 2 and 3 RCT for ‘in trial’ and future accruals from 40 (as in present programme) to 46 months. We would also introduced a 36 month time point. The participants will finish all their sessions (education and maintenance) and we would like to capture this data at this point as opposed to 4 months after completing the intervention at 40 weeks. Therefore time points would be changed to 6, 12, 24, 36, 40 and 46. |
